# Lossed in translation: an off-the-shelf method to recover probabilistic beliefs from loss-averse agents

**DOI:** 10.1007/s10683-015-9429-0

**Published:** 2015-04-17

**Authors:** Theo Offerman, Asa B. Palley

**Affiliations:** 1 CREED, University of Amsterdam, Roetersstraat 11, 1018 WB Amsterdam, The Netherlands; 2 The Fuqua School of Business, Duke University, 100 Fuqua Drive, Box 90120, Durham, NC 27708 USA

**Keywords:** Scoring rule, Subjective probability assessment, Loss aversion, Prospect theory, C81, C91, D03, D81

## Abstract

Strictly proper scoring rules are designed to truthfully elicit subjective probabilistic beliefs from risk neutral agents. Previous experimental studies have identified two problems with this method: (i) risk aversion causes agents to bias their reports toward the probability of $1/2$, and (ii) for moderate beliefs agents simply report $1/2$. Applying a prospect theory model of risk preferences, we show that loss aversion can explain both of these behavioral phenomena. Using the insights of this model, we develop a simple off-the-shelf probability assessment mechanism that encourages loss-averse agents to report true beliefs. In an experiment, we demonstrate the effectiveness of this modification in both eliminating uninformative reports and eliciting true probabilistic beliefs.

**Electronic supplementary material:**

The online version of this article (doi:10.1007/s10683-015-9429-0) contains supplementary material, which is available to authorized users.

## Introduction

Accurately obtaining subjective probabilistic information about uncertain future events is an essential step in the decision making process in many different economic and public policy settings. In many cases, rather than trying to build a model to estimate probabilities, the best and most informative assessments come from an agent who has a good amount of relevant experience and can use her collected wisdom to estimate a subjective probability. Eliciting this information presents an important and difficult problem in many fields such as finance and macroeconomics (Diebold and Rudebusch 1989; Ghysels 1993), decision analysis (Keeney 1982), and meteorology and weather forecasting (Murphy and Winkler 1984). In addition, probability assessments often comprise an important component of economic experiments. Even when the ultimate objective is not to elicit subjective beliefs, obtaining this information may be a critical secondary step in an experimental procedure.

Well-designed scoring rules provide a useful tool for procuring this subjective information by providing an agent with the right incentives to carefully evaluate and quantify her beliefs, and to honestly reveal her subjective assessment of the likelihood of these uncertain future events. The quadratic scoring rule (QSR), a variant of which was first introduced by Brier (1950), is the most commonly used.^1^

The incentive design of scoring rules implicitly assumes, however, that the agent is risk neutral, which contrasts with how people often behave. Winkler and Murphy (1970) examine the effects of nonlinear utility on the optimal report under a proper scoring rule, showing that risk aversion leads an agent to hedge her reports away from categorical forecasts of 0 and 1 and risk seeking leads the agent to bias her reports closer to 0 or 1. This biasing effect of risk preferences can be easily corrected by applying the inverse utility function to the scoring rule (Winkler 1969). In practice, however, an even more troubling pattern of excessive reports equal to the baseline probability of 1/2 emerges as well, a phenomenon not explained by classical expected utility models. For example, Offerman et al. (2009) tested responses by 93 subjects to a QSR for objective probabilities that ranged from 0.05 to 1 and found that they reported 1/2 more than three times as often as they should have (15.3 % versus 5 %). This particular type of conservatism inhibits the decision maker’s ability to discern among a broad domain of moderate beliefs and conceals a significant amount of useful information.

In this paper, we employ the insights of prospect theory (Kahneman and Tversky 1979; Tversky and Kahneman 1992) to understand the ways in which an agent will distort her report when she receives an uncertain reward from a QSR. Employing Palley’s (2015) model of prospect theory with an endogenous reference point, we highlight how loss aversion can account for why an agent may both report 1/2 for a range of moderate beliefs and bias her reports toward 1/2 for beliefs closer to 0 or 1.

The main contribution of our paper is the introduction of a generalized asymmetric QSR, the * L*-adjusted rule, which eliminates the incentives for conservative reports and enables the elicitation of true probabilistic beliefs. The payoffs in this * L*-adjusted rule are the same as in a classical QSR except negative outcomes are shrunk by a factor of * L*, a parameter that controls the size of the loss adjustment. We use previous experimental work estimating population parameters to derive an off-the-shelf variant of this * L*-adjusted QSR that requires no prior agent-specific calibration. In an experiment, we demonstrate its effectiveness in recovering truthful and precise probability assessments, and show that it alleviates the shortcomings associated with the classical QSR. In agreement with previous results, we find that in response to the classical QSR, agents tend to report the implicit benchmark probability of 1/2 for a wide range of beliefs near 1/2 in order to ensure a certain payoff. By matching the choice of *L* to previous empirical estimates of parameters for the overall population, we also obtain a modified QSR that recovers truthful beliefs experimentally. In doing so, we provide a practical and simple off-the-shelf scoring rule that encourages agents to report their beliefs truthfully.

We want to emphasize that the use of the *L*-adjusted QSR is as easy as the use of a standard QSR. Exactly as with a standard QSR, each subject receives a table that lists how their payoff varies depending their probability judgment and the actual outcome of the predicted phenomenon. The only difference between a standard QSR and an * L*-adjusted QSR is that the actual payoffs in the table are changed to accommodate subjects’ loss aversion. As a result, subjects are encouraged to automatically report judgments that are very close to true objective probabilities.

The simplicity of our approach depends to a large extent on the fact that we provide each subject with the same * L*-adjusted QSR based on parameter estimates for the general population. A natural question is how much precision is sacrificed by ignoring differences that may exist in people’s loss-aversion attitudes. To investigate this question, we include a treatment in which we adjust the scoring rule separately for each subject on the basis of an individually estimated loss parameter. Interestingly, we do not find better results for this treatment.

Recently, several related approaches have been suggested to recover true beliefs from conservative reports. Offerman et al. (2009) propose a revealed preference technique that allows the researcher to correct the reported beliefs of agents who are scored according to a standard QSR. In this method, agents initially provide reports for a range of objective probabilities, which then yields an optimal response mapping that can be inverted and applied to infer subjective beliefs from later reports. In an experiment, Offerman et al. demonstrate the effectiveness of this approach in recovering beliefs from reports that do not equal the baseline probability of 1/2. Kothiyal et al. (2011) extend this method to overcome the problem of discriminating between moderate beliefs in a range around the baseline probability of 1/2, for which agents give the same optimal report. By adding a fixed constant to one of the QSR payoffs, they both eliminate the excess of uninformative baseline reports and yield an invertible response mapping that makes possible the recovery of true beliefs, while maintaining the properness of the original scoring rule. Kothiyal, Spinu, and Wakker do not provide an experimental test of their method.

The approaches taken in Offerman et al. (2009) and Kothiyal et al. (2011) are precise and elegant because they do not need to make structural assumptions on how people make decisions under risk. The downside of these methods is that they are laborious to employ, because a sufficiently dense risk-correction map has to be derived for each agent before any inferences can be made. In both decision analysis and many experimental economics applications, the elicitation of beliefs is a secondary goal, and a simpler and quicker approach may be preferred, as long as it does not sacrifice precision. The method presented in this paper pursues this purpose.

Other elicitation methods that do not make use of scoring rules exist as well. For example, if the utility function is unknown, Allen (1987) presents a randomized payment method that relies on the “linearization” of utility through conditional lottery tickets to incentivize truthful reports. Alternatively, Karni (2009) proposes a procedure with two fixed prizes where the payment function is determined by comparing the agent’s report to a random number drawn uniformly from [0,1], analogous to the Becker et al. (1964) mechanism. Under this method, if the agent exhibits probabilistic sophistication, she has a dominant strategy to report her true belief, irrespective of her risk attitudes. However, in experiments, subjects have been found to have a hard time understanding Becker-DeGroot-Marschak-type procedures (Rutström 1998; Plott and Zeiler 2005; Cason and Plott 2012), and empirical comparisons of these methods with scoring rules have yielded mixed results (Hao and Houser 2010; Hollard et al. 2010; Trautmann and van de Kuilen 2011).

The rest of the paper is organized as follows: Sect. 2 introduces our * L*-adjusted QSR and characterizes the corresponding optimal reporting policy under the prospect theory model of risk behavior. We discuss how this predicted behavior provides a parsimonious explanation of previously observed conservative reporting patterns and how the parameter * L* can be calibrated to allow for the recovery of estimates of any probabilistic belief. Readers who are interested mainly in how well our method encourages subjects to simply report true probabilities may skim Sect. 2 and refer to Proposition 1 and Corollary 1. Sections 3 and 4 detail the experiment that we carried out to test the usefulness of this adjusted scoring rule in practice and demonstrate its improvements over the classical QSR. Section 5 concludes and Appendix 1 characterizes reporting behavior for the general asymmetric * L*-adjusted QSR and contains proofs of all results. Appendix 2 in Supplementary Material provides images and instructions from the experimental interface.

## The model

We consider an agent who must report a subjective belief about the chances of an uncertain future event $A$. Her true belief is that event $A$ will occur ($X=1$) with probability $p$ and its complement ${\bar{A}}$ will occur ($X=0$) with probability $1-p$. She submits a reported probability $r \in [0,1]$ that $A$ will occur and receives a payoff according to an * L*-adjusted QSR, a generalization of the asymmetric QSR introduced by Winkler (1994).

### **Definition 1**

(*L*-*adjusted Quadratic Scoring Rule*) The * L*-adjusted asymmetric QSR is defined by

$$\begin{aligned} S_L(X,r) = \left\{ \begin{array}{ll} \frac{(1-c)^2-(1-r)^2}{c^2 L} & {\text{if }}\; {A} \; {\text{ occurs \; and }}\;r < c, \\ \frac{c^2-r^2}{c^2} & {\text{if }}\; \bar{A} \; {\text{ occurs \; and }}\;r < c,\\ \frac{(1-c)^2-(1-r)^2}{(1-c)^2} & {\text{if }}\; {A} \; {\text{ occurs \; and }}\; r \ge c, \\ \frac{c^2-r^2}{(1-c)^2 L} & {\text{if }}\; \bar{A} \; {\text{ occurs \; and }}\; r \ge c. \end{array}\right. \end{aligned}$$

In general, the * L*-adjusted QSR can be centered around any baseline probability $c$ of the event $A$ occurring,^2^ but for most of the paper we will focus on the typical case of a symmetric baseline $c=1/2$. When $L=1$ this scoring rule reduces to the asymmetric QSR and when $L=1$ and $c=1/2$ it reduces to the classical binary QSR.

The pattern of reporting behavior that previous studies have observed cannot be explained by classical expected utility theory. Therefore, to understand how an agent will respond to this risky payoff function, we apply a prospect theory model of risk preferences. Prospect theory applies psychological principles to incorporate several important and frequently observed behavioral tendencies into the neoclassical expected utility model of preferences. This more flexible formulation provides a useful descriptive model of choice under risk (Camerer 2000) and generally includes four main behavioral components:

1. *Reference Dependence* The agent evaluates outcomes as differences relative to a reference point rather than in absolute levels.
2. *Loss Aversion* Outcomes that fall below the reference point (“losses”) are felt more intensely than equivalent outcomes above the reference point (“gains”).
3. *Risk Aversion in Gains, Risk Seeking in Losses, and Diminishing Sensitivity to Both Gains and Losses* The agent tends to prefer a sure moderate-sized outcome over an equal chance of a large gain or zero gain, but prefers an equal chance of taking a large loss or avoiding the loss altogether over a sure moderate-sized loss. In addition, the marginal effect of changes in the outcome for the agent diminishes as the outcome moves away from the reference point.
4. *Probability Weighting* The agent overweights probabilities close to 0 and underweights probabilities close to 1.

Of critical importance in applying prospect theory to model choices under risk is the determination of the reference point. Often the reference point is implicitly set to equal 0, but this assumption may not be realistic when all outcomes are positive, as is typical in practice when rewarding subjects for providing reports according to a QSR. For example, if the reference point were taken to be 0, then all outcomes in our experiment would be viewed as “gains” and the prospect theory model would be unable to explain the observed reporting behavior.

Instead, we argue that even in settings where all outcomes are nominally positive, an agent may still feel elation or disappointment based on whether the payoff she receives falls above (a “gain”) or below (a “loss”) what she expected at the time that she submitted her report. To model this, we assume that the agent possesses a reference-dependent utility function of the form of Palley (2015), in which the agent develops an expectation $E$ about her outcome $S$ from the scoring rule, and this expected outcome then forms a natural reference point for her to evaluate the outcome that she ultimately receives. This utility function extends existing models of an endogenously determined reference point (see, e.g., Shalev (2000)) to accommodate the case of an agent with prospect-theory-type preferences. This model will provide a parsimonious explanation for the behavior that is observed, and most importantly, can be readily used to provide a solution to the problem and insight into why it works.

Specifically, we assume that when the agent’s outcome exceeds this expectation, she feels an additional gain of $(S-E)^\alpha$, where $\alpha \in (0,1]$ specifies the curvature of her risk preferences. When her outcome falls below her expectation, she perceives this as an additional loss equal to $- \lambda (E-S)^\alpha$, where $\lambda \ge 1$ additionally parameterizes the agent’s degree of loss aversion. Mathematically, this utility function is specified by

$$\begin{aligned} v(S,E) = \left\{ \begin{array}{ll} E - \lambda (E-S)^\alpha \quad  {\text{if}}\; S < E \\ E + (S-E)^\alpha \quad {\text{if}}\; S \ge E. \end{array}\right. \end{aligned}$$

If $\alpha =1$, this formulation coincides with the loss-averse utility function detailed in Shalev (2000). If $\alpha =\lambda =1$, then this simplifies to the risk-neutral objective of maximizing expected payoff that the definition of a proper scoring rule implicitly assumes.^3^

In addition, we assume that the agent applies probability weighting functions $w_+(p)$ and $w_-(p)$ for scores that fall above and below $E$ (positive and negative events), respectively. $w_+(\cdot )$ and $w_-(\cdot )$ are assumed to be strictly increasing with $w_+(0) = w_-(0) = 0$, $w_+(1) = w_-(1) = 1$, and $w_+(p)+w_-(1-p)=1$ for all $p \in [0,1]$.^4^ The *ex ante* expected-valuation that an agent receives from responding to a binary scoring rule is then given by a probability-weighted sum over the possible scores; $V(E) = \sum _S w(p_S) v(S,E).$ 5

**Fig. 1:**
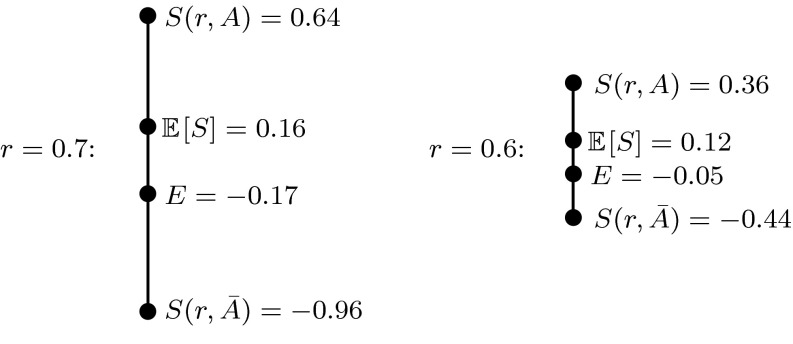
Two examples of an agent’s possible report choices and corresponding *ex ante* reference point formation in response to an classical QSR with baseline $c=0.5$ when the agent believes the probability of event $A$ is $p=0.7$, has prospect theory parameters $\lambda =2.4$ and $\alpha =1$, and does not apply probability weighting

Until this point, we still have not specified the details of how the reference point $E$ is determined. The motivating intuition we follow here is that the agent’s expected-valuation of the prospect should be consistent with her expectation about the prospect. In other words, if she uses $E$ as her reference point in determining $V(E)$, then the resulting expected-valuation should simply equal $E$ itself. Specifically, we assume that the reference point $E$ is determined endogenously according to the consistency equation $V(E)=E$, as in Palley (2015). In this sense, for a given prospect, a consistent reference point $E$ is the expectation that perfectly balances the agent’s potential gains against her potential losses, weighted according to her beliefs of their respective likelihoods.

A consistent reference point $E$ is the natural evaluation of a prospect for an agent who carefully contemplates the possible outcomes and anticipates her possible *ex post* feelings, providing a summary measure of how the agent evaluates the risk in an *ex ante* sense. An agent who initially forms a reference point $R$ higher than $E$ will find that her expected losses $-\lambda (R-S)^\alpha$ outweigh her expected gains $(S-R)^\alpha$, causing her to adjust her expectation downward.

Conversely, an agent whose reference point is initially lower than $E$ will find that her expected gains outweigh her expected losses, causing her adjust her reference point upward. A thoughtful agent will thus converge to a unique consistent expectation $E$. This notion of expectations as an endogenously determined reference point is introduced and developed in the models of Bell (1985), Loomes and Sugden (1986), Gul (1991), Shalev (2000), and Koszegi and Rabin (2006, 2007).

Note that the relationship between the reference point and the valuation function possesses an intentional “circularity,” which is an important part of the model. For any prospect, there exists only one unique reference point *E* that satisfies *V*(*E*) = *E*, and this is the reference point that represents the agent’s ex ante valuation of a given prospect. It is this equation that ensures the consistency of the valuation function and the reference point, and which pins down the appropriate expectation *E*.

**Fig. 2:**
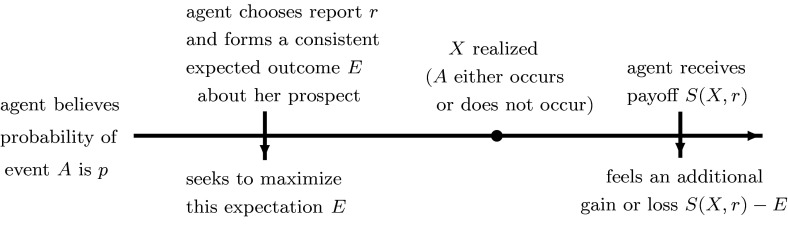
Timeline of the agent’s report choice, reference point formation, and ex post evaluation of the event

Figure 1 displays an example of this reference point formation process. We see that a loss-averse agent with subjective beliefs of $p=0.7$ would derive an *ex ante* expectation of $-0.17$ from truthfully reporting $r=0.7$ in response to a QSR, while deriving an *ex ante* expectation of $-0.05$ from reporting $r=0.6$. Both of these reports are therefore dominated by reporting the baseline $r=0.5$, which yields an outcome of 0 with certainty and a corresponding *ex ante* expectation of $0$. Whereas a risk-neutral agent would prefer to report $r=0.7$, which yields the highest expected score, the loss-averse agent in this case will prefer to report $r=0.5$.

We assume that the agent seeks to maximize her expected outcome $E$ over all possible reports $r \in [0,1]$, subject to the consistency requirement, which essentially means that the agent will consider her *ex post* prospects when she chooses her report and forms her *ex ante* expectation about her outcome. The timeline of events is displayed in Fig. 2.

### **Proposition 1**

*The optimal consistent report function when* $c=0.5$ *is given by*

$$\begin{aligned} r_L^*(p) = \left\{ \begin{array}{ll} \frac{ \varLambda (p)^{\frac{1}{\alpha}} }{ \varLambda (p)^{{\frac{1}{\alpha }}} + L }, & p < \min \left\{ w_-^{-1} \left( \frac{L^{\alpha }}{\lambda +L^{\alpha }} \right) , \frac{1}{2} \right\} \\ \frac{1}{2}, & \min \left\{ w_-^{-1} \left( \frac{L^{\alpha }}{\lambda +L^{\alpha }} \right) , \frac{1}{2} \right\} \le p \le \max \left\{ w_+^{-1} \left( \frac{ \lambda }{ L^\alpha + \lambda } \right) , \frac{1}{2} \right\} \\ \frac{ L }{ \varLambda (1-p)^{{\frac{1}{\alpha }}} + L }, & p > \max \left\{ w_+^{-1} \left( \frac{ \lambda }{ L^\alpha + \lambda } \right) , \frac{1}{2} \right\} , \end{array}\right. \end{aligned}$$

*where* $$\varLambda (p) = \frac{ \lambda w_-(p) }{w_+(1-p)}$$ *is the agent’s loss-weighted odds ratio of event* $$A. $$

The optimal consistent response function for more general (asymmetric) baseline probabilities $c$ can be found in Appendix 1.^6^

### **Proposition 2**

*For any positive linear rescaling of the payoffs* $\tilde{S}_L(r) \equiv a S_L(r) + b$, $a>0, b \in {\mathbb{R}}$, *the optimal consistent report remains* $\tilde{r}_L^*(p) = r_L^*(p)$ *and the corresponding optimal* *ex ante* *expected outcome is rescaled according to* $\tilde{E}^*(p) = a E^*(p) +b$.

In other words, in contrast to the predictions of the cumulative prospect theory model with a fixed reference point and many classical utility formulations, the agent’s behavior will be invariant to positive linear rescaling of the payoffs. This means, for example, that the agent’s optimal behavior would not change if the decision maker decided to pay her in a different currency with exchange rate $a$:1 or pay her an additional fixed fee $b$ for providing the report.

**Fig. 3:**
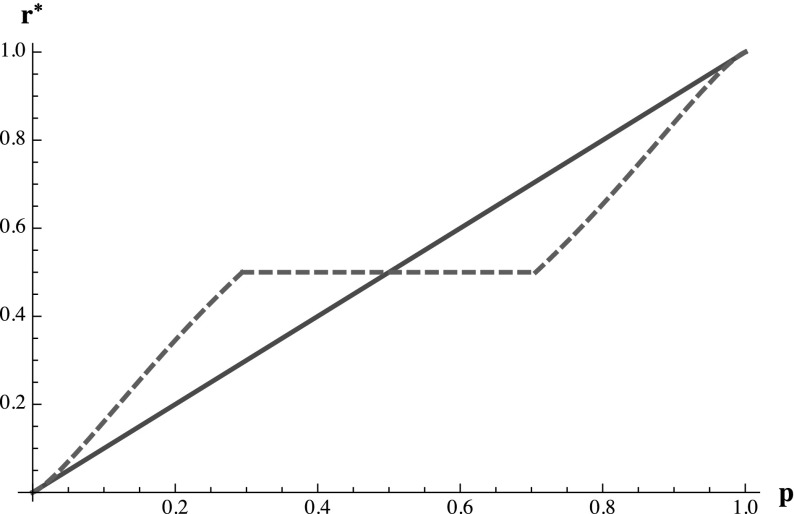
Optimal consistent report $r^*(p)$ (the *dashed line*) to the classical QSR ($c=0.5$, $L=1$) for $\lambda =2.4$, $\alpha =0.8$, and $w_-(p)=w_+(p)=p$ versus truthful reporting (the *solid line*)

Figure 3 displays the shape of optimal reports as a function of the agent’s beliefs $p$ in response to the classical QSR. For a large region of moderate beliefs near $1/2$, the agent will prefer to simply report $1/2$ in order to receive a payoff of $0$ with certainty. While the width of this region depends jointly on $\lambda$, $\alpha$, $w_+(\cdot )$, and $w_-(\cdot )$, it is largely driven by the loss aversion parameter $\lambda$. The shape of the optimal consistent report function closely mirrors the theoretical results of Offerman et al. (2009). Here the decision maker cannot simply provide the agent with the classical QSR and then infer her true beliefs from her report because the resulting response function $r^*(p)$ is not invertible. All beliefs $p$ in the interval $[w_-^{-1} \left( \frac{1}{\lambda +1} \right) \le p \le w_+^{-1} \left( \frac{\lambda }{\lambda +1} \right) ]$ are mapped to the conservative risk-free report of $1/2$ (this is the flat region of the optimal report function). This means that observing a report of $1/2$, which may happen quite frequently if the agent is loss-averse and has moderate beliefs, tells the decision maker only that the agent’s beliefs lie somewhere within that interval.

### Determining the $L$-adjustment

To recover true beliefs, the decision maker needs to instead adjust the scoring rule to eliminate the “flat region” of conservative reports of $1/2$, which will allow him to invert the agent’s optimal report function $r^*(p)$ and estimate $p$ according to $r^{*-1}(r)$. Sensitivity analysis suggests that loss aversion accounts for the largest proportion of this conservative behavior. The best way to counteract this phenomenon, then, is to adjust the scoring rule so that negative outcomes are less severe by a factor of $\frac{1}{L}$. By computing the value $L^*$ that solves

1 $$\begin{aligned} w_-^{-1} \left( \frac{L^{\alpha }}{\lambda +L^{\alpha }} \right) = w_+^{-1} \left( \frac{ \lambda }{ L^\alpha + \lambda } \right) , \end{aligned}$$

the decision maker can squeeze the endpoints of the “flat region” of conservative reports of $1/2$ together and recover the agent’s true beliefs.

#### **Corollary 1**

*The optimal adjustment when* $c=0.5$ *is given by*

$$\begin{aligned} L^* = \varLambda (1/2)^{1/\alpha } = \left( \frac{\lambda w_-(1/2)}{w_+(1/2)} \right) ^{1/\alpha }. \end{aligned}$$

This calibration of $L = L^*$ eliminates the agent’s incentive to provide these uninformative reports even for very moderate beliefs close to $1/2$, and also removes almost all of her distortion in the optimal reporting function. After receiving her report, the decision maker can apply the inverse of the optimal report function to the observed report $r$ to recover the agent’s exact truthful beliefs $p = r_L^{*-1}(r)$. In the absence of utility curvature and probability weighting ($\alpha =1$ and $w(p)=p$), the optimal adjustment is simply equal to the loss aversion parameter ($L^* = \lambda$) and the inversion step is unnecessary because the optimal report function is truthful ($r_\lambda ^*(p)=p$).

In practice, an agent’s report may include a noisy error term $\epsilon$, so that the agent reports $r_L^*(p) + \epsilon$ instead. This means that the inferred beliefs will also contain an error of $r_L^{*-1}(r_L^*(p) + \epsilon ) - r_L^{*-1}(r_L^*(p)).$ However, since $r_L^{*-1}(\cdot )$ is differentiable and close to the identity function for a broad range of reasonable parameter values, the resulting error in inferred beliefs simply scales roughly equally to the size of the original reporting error. Another concern with the * L*-adjustment method is that it may become laborious if agents are very heterogeneous. In such a setting, the model parameters $\alpha$, $\lambda$, and $w(p)$ and the corresponding $L^*$ would have to be estimated individually. Our experimental results show, however, that heterogeneity is only of secondary importance and that our method does a remarkable job even without a correction of individual differences.

**Fig. 4:**
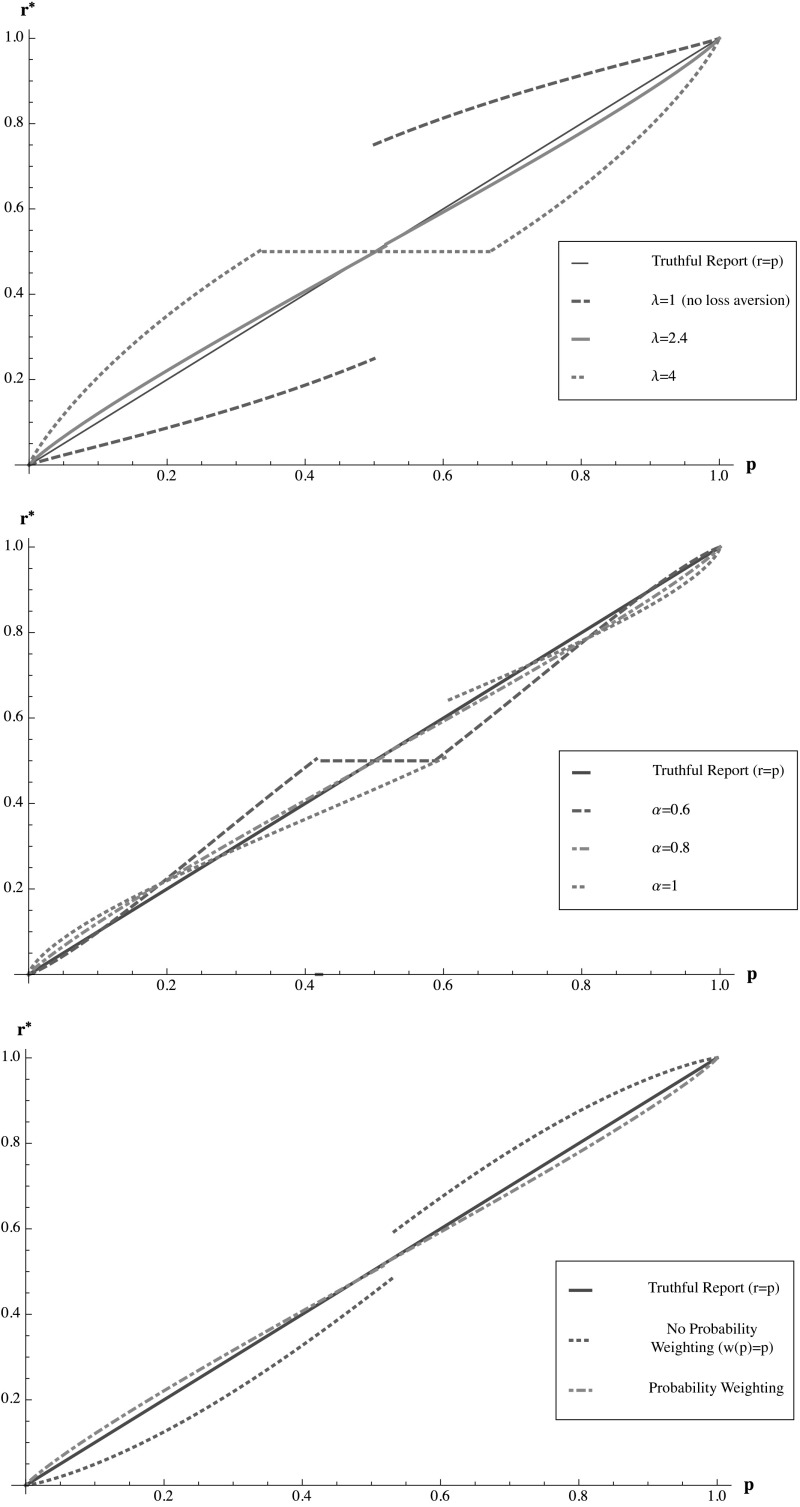
Optimal consistent reports $r_L^{*}(p)$ in response to the * L*-adjusted QSR with $L=3.7$ and $c=0.5$ when $\lambda =2.4$, $\alpha =0.8$, $\delta _+ = 0.8$, $\gamma _+ = 0.7$, $\delta _- = 1.1$ and $\gamma _- = 0.7$ versus truthful reporting (the *solid line*). The upper graph considers varied values of $\lambda$, keeping the other parameters fixed. The *middle graph* considers varied values of $\alpha$, keeping the other parameters fixed. The *lower graph* considers the cases of probability weighting and no probability weighting, keeping the other parameters fixed

Figure 4 displays the optimal reports in response to an * L*-adjusted scoring rule, which is calibrated to average parameter estimates $\lambda =2.4$, $\alpha =0.8$, $\delta _+ = 0.8$, $\gamma _+ = 0.7$, $\delta _- = 1.1$ and $\gamma _- = 0.7$ (yielding $L^*=3.7$) from the studies discussed in footnotes 4 and 5 for the general population, for an agent with various actual loss aversion parameters $\lambda$, utility curvature parameters $\alpha$, and both with and without probability weighting. As might be expected, given that the adjustment is primarily designed to address distortions due to loss aversion, optimal report functions are most sensitive to misestimation of the parameter of loss aversion $\lambda$, and are less sensitive to variations in $\alpha$ and the probability weighting functions. This suggests that if the decision maker does not want to assess individual parameters, the most important measurement to focus on is $\lambda$. We also see that if $L^*$ is miscalibrated due to errors in parameter estimates, he may observe reports both above and below the true beliefs $p$, depending on whether the $\lambda$ estimate is too high or too low.

Next, note that any remaining difference between the optimal report function in response to the $L^*$-adjusted rule and truthful reporting, which in theory could be corrected by applying $r^{*-1}(\cdot )$ to the observed report, would be completely swamped by any noise in reports and the distortionary effects of errors in the parameter estimates. As a result, in practice there is very little benefit to attempting to carry out this second inversion step on the reports $r$. A more practical approach is to simplify the assessment process by eliminating this second inversion step and taking the reported probability as our estimate of the agent’s true beliefs. In doing so, the decision maker should keep in mind the remaining potential for distortion, which is mainly caused by incorrect estimation of the agent’s parameters, and understand that her reports may be somewhat noisy due to this miscalibration.

If the decision maker wishes to avoid the potentially laborious process of individually assessing parameter values for each agent beforehand, a simple approach is to simply present the agent with the * L*-adjusted QSR with $L^*=3.7$ and take her resulting report as the estimate of her true beliefs. If the decision maker does want to spend some time and effort to estimate the agent’s parameter values ahead of time, he should focus on accurately assessing her loss-aversion parameter $\lambda$, since this offers a fair amount of flexibility in calibrating the scoring rule, and variation in the other parameter values has a less significant effect on the optimal report function.

Below we outline a simple approach that the decision maker can use to estimate $\lambda$ and $L$ on an individual basis: first, assume that the agent’s utility curvature is $\alpha =1$ and probability weighting function is $w(p)=p$. This implies that for a 50–50 lottery between receiving $x_1$ and $x_2$, where $x_1 \le x_2$, her consistent expectation is given by^7^

2 $$\begin{aligned} E = ( x_2 + \lambda x_1 ) / ( 1 + \lambda ). \end{aligned}$$

Next, the agent is offered a choice from a carefully designed set of coin flips that offer different payoffs depending on whether the coin ends up heads or tails. We assume that the agent makes this choice so that she maximizes the consistent expectation given in Eq. 2, meaning that she will prefer different lotteries for different values of $\lambda$. Specifically, the set of coin flips offered is designed so that each lottery is the most preferred option for a specific interval of possible $\lambda$ values. Once the agent selects her most preferred lottery from this set, the decision maker can use her choice to make inferences about her loss aversion parameter, for example, by taking the midpoint of the interval of $\lambda$ values for which that flip is the most preferred. As noted in the discussion of Corollary 1, under these assumptions, the optimal $L$-adjustment is then simply equal to that $\lambda$ estimate. An example of such a set of coin flip lotteries and the $\lambda$ parameters implied by each can be found in the description of the IC treatment in Sect. 3.

## Experiment

Offerman et al. (2009) show that, in practice, proper scoring rules fail to elicit truthful reports from human agents, with patterns of reporting behavior that match the theoretical model of this paper. In this experiment we confirm the predictions of the preceding theory and demonstrate the feasibility of the * L*-adjusted scoring rule in recovering truthful beliefs from human subjects. In doing so, we demonstrate that the * L*-adjusted rule provides a simple modification of the QSR that can be used for most agents to obtain relatively accurate reports from the general population without having to arduously assess individual parameter and curvature estimates. In addition, we test several values of $L$ and show that the proposed rescaling $L^*=3.7$ is indeed the most effective at eliciting truthful beliefs from agents.

### Experimental design and procedures

The computerized experiment was carried out at the CREED laboratory of the University of Amsterdam. Subjects were recruited from the undergraduate population using the standard procedure, with a total of 183 subjects participating in the experiment. Subjects earned on average 13.00 euros (€) for an experiment that lasted approximately 35 min. Subjects read the instructions on their screen at their own pace. After finishing the instructions, they had to correctly answer some control questions that tested their understanding before they could proceed to the experiment. Subjects also received a handout with a summary of the instructions before beginning the experiment (Appendix 2 in Supplementary Material provides a sample of the instructions).

We employed a between-subjects design, in which each subject participated in exactly one of four treatments. The first three treatments differed only in the size of the loss correction applied to the QSR. In the control treatment we used $L=1$, which therefore corresponds to the classical QSR that has been previously employed in many experiments. We refer to this treatment as NC (mnemonic for no correction). In treatment medium correction (MC), we applied a moderate-sized correction of $L=1.5$ and in treatment large correction (LC) we applied the large loss correction of $L=3.7$ derived and predicted to be optimal in Sect. 2.1.

In each of these three treatments, subjects were informed that the experiment would last for 20 rounds and that at the end of the experiment one of the rounds would be randomly selected and used for actual payment. In each round, a subject was asked to give a probability judgment that a randomly drawn number from the set $\{0, 1, \dots , 99, 100\}$ would be in the range $\{0, 1, \dots , Y\}$. The randomly drawn number was an integer and subjects knew that each number in the set $\{0, 1, \dots , 99, 100\}$ was equally likely. The range was given at the start of a round and differed across rounds. The lower bound of the range was 0 and the upper bound, which determined the true objective probability, differed across rounds. In the 20 rounds we used the $Y$ values $\{5, 10, \dots , 30, 33, 35, 40, \dots , 95 \}$, in a random order. For example, in the round that used $Y=45$, the subject was asked to give the probability judgment that the randomly drawn integer would fall in the set $\{0, 1, \dots , 45\}$. While the subject was free to report any probability that he or she wanted, the objective probability of this event is given by $p=\frac{Y+1}{101}$, so in the example of $Y=45$ the true probability was $p=\frac{46}{101}$. Each subject was presented with the ranges in a random order to prevent the possibility that order effects might confound the results. Subjects did not receive any feedback between successive rounds, so there was no opportunity to learn from previous rounds.

Subjects were given a handout with a tabular depiction of the * L*-adjusted QSR that pertained to their treatment. The table clarified how their possible payoffs would change depending on what probability they reported. The scoring rules were in units of euros rescaled by a factor of 3 and shifted upward by 12, so that payments ranged between a minimum of €3 and a maximum of €15, and participants could assure themselves a payoff of €12 by always reporting $r=0.5$.

Appendix 2 in Supplementary Material includes the three payoff tables that we used in the experiment. When a subject had tentatively decided which report $r$ he or she wanted to provide in a given round, they were asked to type this probability judgment into a box on the upper part of the screen. Once this response was entered, the lower part of the screen then automatically displayed the relevant part of the payoff table with the current decision highlighted. Using arrows, subjects could scroll through the payoff table and if they desired, increase or decrease their report until they settled upon an ultimate response. Their choice was not finalized until they clicked the button “Satisfied with choice” (Appendix 2 in Supplementary Material shows the decision screen). After a subject had provided all 20 responses, the computer randomly selected exactly one round (indexed by the upper bound of its range $Y$), which then determined his or her payment as follows: first, the computer drew a random integer from the set $\{0, 1, \dots , 99, 100\}$ and determined whether the number was in the range $\{0, 1, \dots , Y\}$ of that round or not. Second, the payoff was determined by inputting both the realization of whether the number was in the range or not and the subject’s probability judgment $r$ for that round into to the scoring rule that the subject had faced. At the end of the experiment subjects filled out a questionnaire and were privately paid their earnings.

In the fourth treatment we provided each subject with an individually calibrated * L*-adjusted rule.^8^ We included this treatment individual correction (IC) to investigate how much precision was lost by correcting each subject with the same * L*-adjusted QSR. The uniform loss corrections that we use in MC and LC may not work well when subjects differ substantially in their loss-aversion attitudes. Treatment IC consisted of two parts: At the start, subjects were informed that they would make 21 decisions in total, 1 in part 1 and 20 in part 2, and that at the end of the experiment one of these 21 decisions would be selected at random for actual payment. While making their decision for part 1, subjects did not yet have access to the instructions of part 2. In part 1, each subject chose one of the 12 options listed in Table 1. Subjects were told that if this decision were selected for payment, their chosen option would determine their payment together with the outcome of a random coin toss by the computer. If the coin flip came up heads (tails), then the payoff in the second (third) column would apply.

**Table 1:** Individual assessment of the loss-aversion parameter $\lambda$ (part 1 of treatment IC)

Option	Earnings if Heads	Earnings if Tails	Implied L ($\lambda$ interval)	How often chosen?	$|p-r|$
1	24.25	3.00	1.0 ($\lambda \le 1.25$)	7	8.6 (12.1)
2	23.00	4.00	1.5 ($1.25 \le \lambda \le 1.75$)	2	11.6 (10.4)
3	21.25	5.00	2.0 ($1.75 \le \lambda \le 2.25$)	1	24.9 (18.3)
4	19.00	6.00	2.5 ($2.25 \le \lambda \le 2.75$)	13	8.7 (9.9)
5	16.25	7.00	3.0 ($2.75 \le \lambda \le 3.25$)	9	9.4 (13.8)
6	13.00	8.00	3.5 ($3.25 \le \lambda \le 3.75$)	8	6.9 (9.4)
7	11.50	8.40	4.0 ($3.75 \le \lambda \le 4.25$)	7	6.6 (5.8)
8	10.65	8.60	4.5 ($4.25 \le \lambda \le 4.75$)	1	47.3 (29.4)
9	9.65	8.75	5.0 ($4.75 \le \lambda \le 5.25$)	1	10.5 (9.7)
10	9.47	8.84	5.5 ($5.25 \le \lambda \le 5.75$)	0	–
11	9.18	8.89	6.0 ($5.75 \le \lambda \le 6.25$)	0	–
12	8.93	8.93	7.0 ($\lambda \ge 6.25$)	1	1.9 (3.8)

The left 3 columns list the options between which the subjects in part 1 of treatment IC were asked to choose; earnings are denoted in euros. The fourth column lists the * L*-parameter implied by a choice. The fifth column lists how often each option was chosen and the final column lists, for each option, subjects’ average absolute deviations of the reported probabilities $r$ from the true probabilities $p$ (as determined by the range $Y$ according to $p=(Y+1)/101$), with the standard deviations in parentheses

The fourth column of Table 1 lists the * L* parameter implied by a subject’s choice (this was not observed by our subjects). After part 1, subjects proceeded with part 2, which was the same as in the other three treatments, except for the fact that each subject was provided with their own individual * L*-adjusted scoring rule corresponding to their choice in part 1. The 12 possible payoff tables for these * L*-adjusted QSRs are included in Appendix 2 in Supplementary Material.

We ran two separate sessions for each treatment. In total, 45 subjects participated in NC, 42 subjects in MC, 46 subjects in LC, and 50 subjects in IC.

**Fig. 5:**
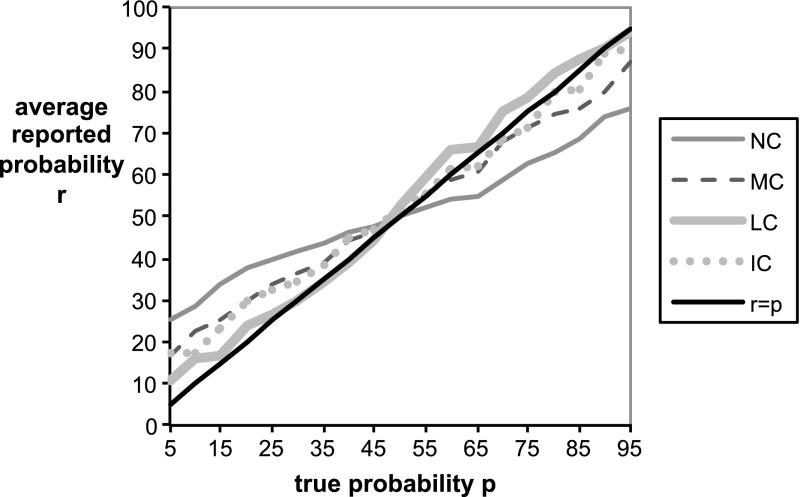
Average reported probability function $r(p)$ for each treatment versus the true objective probability report $r=p$. Note that probabilities in the graph are written in percentage terms (% from 0 to 100) rather than decimal units (0–1)

## Experimental results

We start with a brief description of the individual differences in treatment IC listed in Table 1. In part 1, 74 % of the subjects chose options that correspond to moderate * L* parameters in the range $[2.5, 4]$. The most common deviation was for subjects to behave risk-neutrally and choose option 1; 14 % of our subjects behaved in this way. The final column of Table 1 displays the absolute difference between reported and true probabilities, averaged for all individuals who chose the same option in part 1. Interestingly, there is no clear relation between a subject’s implied * L* parameter and the average absolute deviation of the reports from the true probabilities. Subjects with large loss adjustments can be corrected roughly as well as subjects with small loss adjustments.

**Fig. 6:**
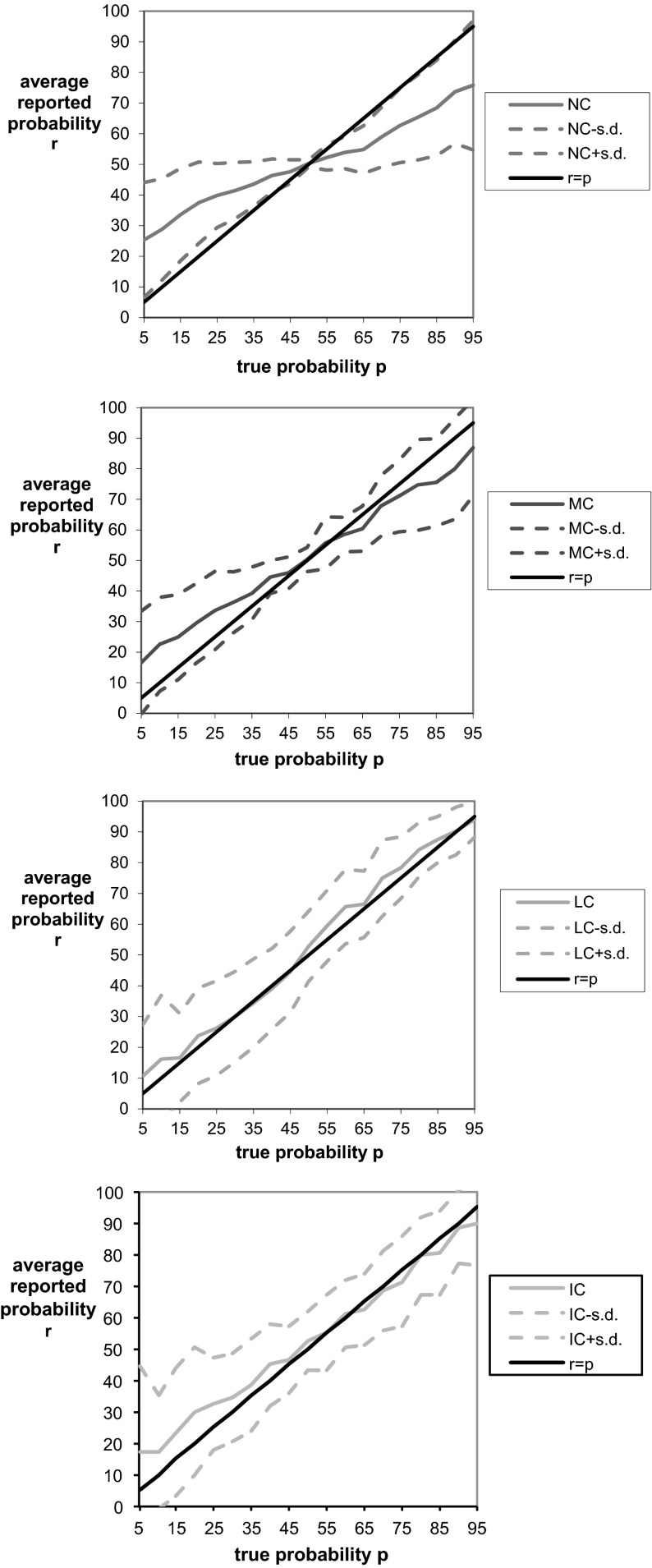
Average reported probability function $r(p)$ with $+/-$ one standard deviation for each treatment versus the true objective probability report $r=p$. Note that probabilities in the graphs are written in percentage terms (% from 0 to 100) rather than decimal units (0 to 1)

**Fig. 7:**
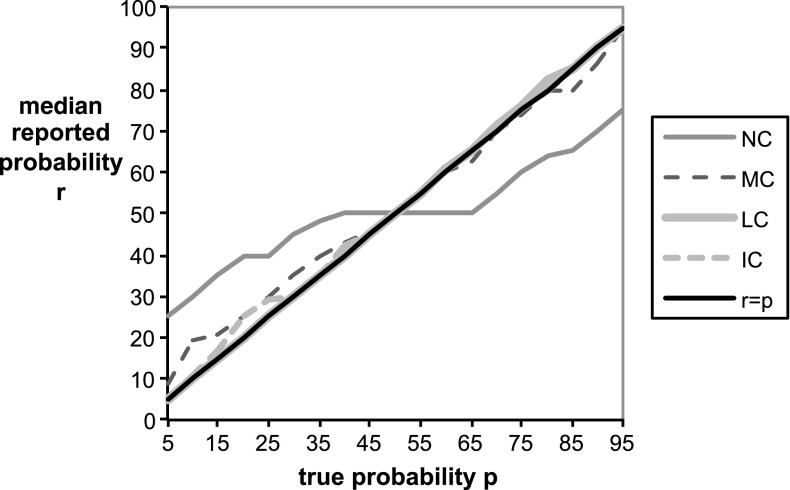
Median reported probability function $r(p)$ for each treatment versus the true objective probability report $r=p$. Note that probabilities in the graph are written in percentage terms (% from 0 to 100) rather than decimal units (0 to 1)

Figure 5 provides an overview of the results by graphing the average reported probabilities in each treatment as a function of the true objective probability $p$. The solid black line presents the ideal report function of correct objective probabilities $r=p$. The control treatment NC displays a commonly observed pattern for data collected with uncorrected scoring rules. Subjects overwhelmingly bias their reports in the direction of risk aversion by reporting probabilities that are closer to 50 % than the true probabilities. In the treatment with a medium correction MC, subjects’ these differences are substantially diminished compared to the control treatment, but a systematic bias in the direction of risk aversion still survives. The treatment with individual corrections IC provides on average the same results as MC when the true probability is below 50 % but better results for true probabilities above 50 %. However, under the treatment with a large loss correction LC, the systematic bias vanishes and the average reported probabilities are almost identical to the true probabilities across the whole range of $p \in [0,1]$.

**Table 2:** Comparison between treatments

Treatment	$$ | p-r |$$	$$(p-r)^2$$	Spearman Rank $$\rho $$	Frequency of 50 % reports	Frequency of 50 % reports for *p* = 45 %, *p* = 55 %	Risk Bias
NC	12.9 (11.8)	305.9 (524.5)	0.75	39.8 %	62.2 %	12.0 (13.3)
MC	8.5 (10.1)	173.7 (358.1)	0.87	23.5 %	39.3 %	5.7 (12.3)
LC	7.9 (10.9)	180.9 (541.2)	0.92	10.1 %	22.8 %	−1.0 (13.5)
IC	9.4 (12.8)	252.0 (833.6)	0.84	16.0 %	24.0 %	3.9 (15.7)
Mann-Whitney probability						
NC versus MC	0.00	0.01	0.02	0.02	0.02	0.00
NC versus LC	0.00	0.00	0.00	0.00	0.00	0.00
NC versus IC	0.01	0.01	0.13	0.00	0.00	0.00
MC versus LC	0.43	0.46	0.58	0.01	0.06	0.00
MC versus IC	0.86	0.88	0.46	0.15	0.10	0.21
LC versus IC	0.33	0.37	0.20	0.26	0.70	0.01

Probabilities in the table are written in percentage terms (% from 0 to 100) rather than decimal units (0–1). Each cell lists the average for the relevant statistics, with the standard deviations in parentheses. $p$ denotes the true probability (determined by the range $Y$ according to $p=\frac{Y+1}{101}$) and $r$ denotes the reported probability. ‘Frequency of 50 % Reports for *p* = 45 %, *p* = 55 %’ denotes the relative occurrence of 50 % reports when the true probability equals 45 or 55 %. Risk bias equals $p-r$ if $p>0.5$, and equals $r-p$ if $p<0.5$ (cases where $p=0.5$ are excluded). Mann-Whitney tests use average statistics per subject as data points (45 subjects in NC, 42 subjects in MC, 46 subjects in LC, and 50 subjects in IC). In the text, the threshold for significance is at the 5 % level

A good elicitation method not only avoids systematic biases but also minimizes variance in the reported probabilities, so that reports will be both honest on average and relatively precise, meaning that a typical deviation from the honest report will not be too large. Figure 6 provides a more detailed view of reports in each treatment by adding standard deviations above and below the average reported probabilities. For treatments NC and MC, the standard deviation is smallest for the true probability of 50 % and increases proportionally with the distance between the true probability and 50 %. The picture is somewhat different for treatments LC and IC, in which the standard deviation gradually diminishes as the probability increases. Figure 7 displays the median reports in each treatment, which provides another perspective of the ‘typical’ behavior under each treatment. We can see that median reports in the control treatment NC display a wide flat region of uninformative reports near $0.5$ that is predicted by the preceding theory. This characteristic flat region, which is highlighted more readily by the computation of the proportion of 50 % reports in Table 2 below, is masked in the graphs in Fig. 6 because the underlying flat region is averaged against more extreme reports.

Table 2 compares the performance of the treatments with respect to six measures. First, for each subject we computed the average absolute difference between the reported and true probabilities. Both treatments MC and LC that apply a loss correction perform substantially better than the control treatment without such a correction, with absolute errors roughly halved. Mann-Whitney tests that use average statistics per subject as data points reveal that the differences between MC and NC and between LC and NC are both significant. Thus, in both treatments where a uniform loss-correction is applied (MC and LC), subjects’ reported probabilities are systematically closer to the actual probabilities than without a loss-correction (NC). Treatment LC performs on average somewhat better than MC, but this difference is not significant. Surprisingly, treatment IC produces on average somewhat worse results than LC and MC, but the differences are far from significant. Like MC and LC, IC yields a clear and significant improvement compared to NC.

A similar picture emerges for our second error measure, which is based on subjects’ average squared differences between reported and true probabilities. Again, the MC, LC, and IC treatments substantially and significantly outperform the control treatment NC, and while LC additionally seems to do a somewhat better job than MC and IC, the latter differences are not significant.

As a third measure, we computed the Spearman rank correlation between reported and true probabilities for each subject. Ideally, a belief elicitation measure would elicit beliefs that perfectly correlate with true probabilities. In studies that employ uncorrected scoring rules, it is well known that a few subjects are very much attracted by the sure payoff corresponding to a report of 50 %, which results in a poor correlation between reported and true probabilities. Table 2 shows that indeed MC and in LC produce substantially and significantly higher Spearman rank correlation coefficients than NC does. Likewise, IC also yields a clearly larger correlation than NC, but this difference fails to reach conventional significance levels. The differences between MC, LC and IC are again insignificant.

As a fourth measure, we compare the treatments to the extent that they induce uninformative 50 % reports. If subjects were to always report true probabilities, reports of 50 % should occur in only $1/20$th of the cases. NC and MC substantially overshoot this ideal benchmark, with frequencies of 50 % reports equaling 39.8 and 23.5 %, respectively. In comparison, IC and in particular LC perform very well, producing such reports only 16.0 and 10.1 % of the time, respectively. All pairwise differences between the treatments are significant with respect to this frequency of 50 % reports, except the one between MC and IC and the one between LC and IC.

The fifth measure focuses on the frequency of uninformative 50 % reports when the true probability equals 45 or 55 %. As explained in Sect. 2, loss corrections are expected to matter most for such true probabilities close to 50 %. In agreement with the theoretical arguments, the difference in the frequency of reports of 50 % is particularly large in this category. NC and MC perform especially poorly with respect to this benchmark, with frequencies of 50 % reports equaling 62.2 and 39.3 %, respectively. Again, LC and IC do a much better job in comparison; in these treatments, such reports occur only 22.8 and 24.0 % of the time, respectively.

Finally, our sixth measure makes precise the extent to which the three treatments suffer from systematic risk biases. For each subject, we computed how much on average a subject biased the report in the direction of 50 %. If the average risk bias is positive (negative) then this provides evidence that subject are risk averse (risk seeking). Consistent with Fig. 6, the final column of Table 2 shows that subjects are very biased in the direction of risk aversion in treatment NC. In treatments LC and IC, there is almost no bias, and the bias in treatment MC falls roughly in the middle of the other treatments. All risk bias differences between the treatments are highly significant, except the one between MC and IC.

**Fig. 8:**
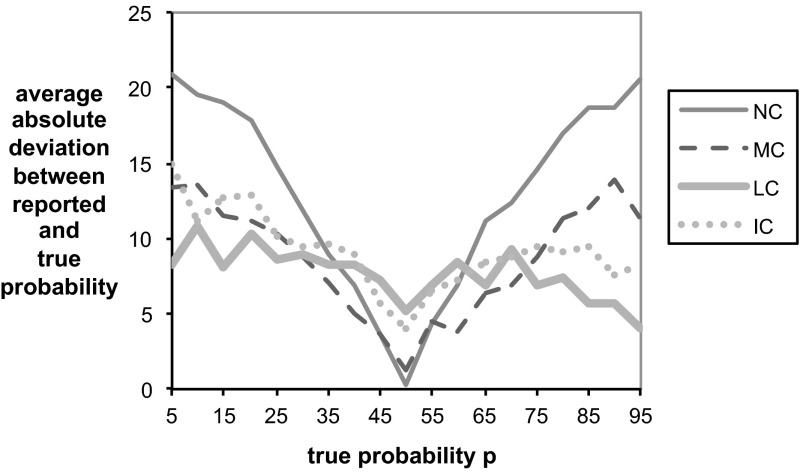
Average absolute error $|r-p|$ in the reported probability function $r(p)$ for each treatment. Note that probabilities in the graph are written in percentage terms (% from 0 to 100) rather than decimal units (0–1)

Figure 8 shows how average absolute errors $|r-p|$ in the report vary with the objective probability $p$ in each of the treatments. The uncorrected scoring rule performs well precisely where we would expect it to—the incentives to make a conservative baseline report of 50 % impel almost unanimously honest reporting when the objective probability is in fact very close to 50 %. However, the uncorrected scoring rule performs far worse than the loss-corrected scoring rules when the true probabilities are larger than approximately 65 % or smaller than approximately 35 %. In other words, errors in the uncorrected scoring rule occur exactly in cases where the effects of loss and risk aversion kick in most heavily. Overall, the uncorrected scoring rule thus proves to be unreliable for eliciting subjective beliefs, since the decision maker does not know which of these regions the true probability belongs to.

**Fig. 9:**
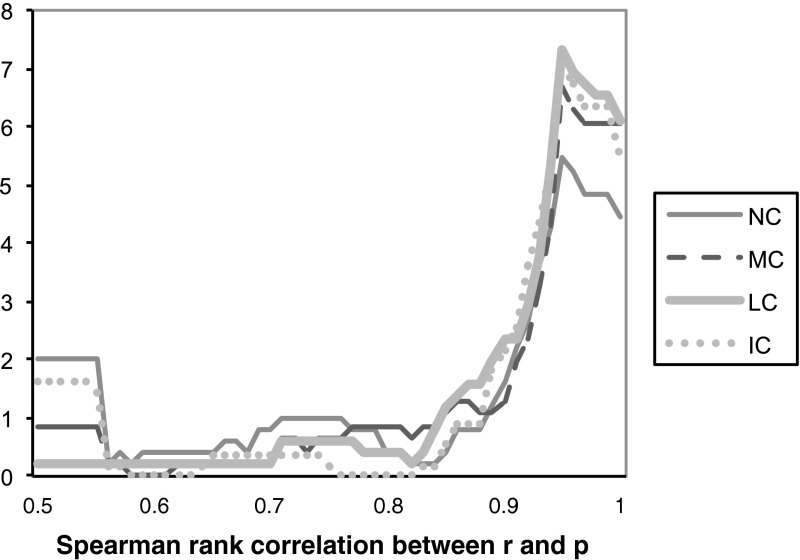
Histogram of the Spearman-rank correlation (SRC) between the true probabilities $p$ and the subject’s reported probabilities $r$. The figure displays for each SRC the percentage of subjects that fall in the interval [SRC − 0.05, SRC + 0.05]. The few observations where SRC < 0.5 are added to SRC = 0.5

Figure 9 displays the empirical density of the Spearman-rank correlation coefficients in the three treatments. In all treatments most subjects have fairly high Spearman-rank correlation coefficients larger than 0.9, while a few subjects have very low coefficients smaller than or equal to 0.5. The treatments differ primarily in the relative frequency of these two categories of correlation coefficient (high or low). The proportion of overly cautious or haphazard reporters with a low coefficient of less than or equal to $0.5$ equals 20.0 % in NC, 14.0 % in IC, 7.1 % in MC and only 2.2 % in LC.^9^

## Discussion

In practice, quadratic and other proper scoring rules can fail to recover the true probabilistic beliefs that they are designed to elicit. Distortions in agents’ reports generally take one of two forms: first, a risk-averse agent may bias her report away from categorical beliefs of $0$ and $1$, as predicted by, for example, the theory of Winkler and Murphy (1970). Second, a risk-averse agent with moderate beliefs close to the baseline probability of $1/2$ may revert to simply reporting $1/2$ in order to receive a risk-free payoff. In other words, under proper scoring rules such as the classical QSR, we should expect to see a large proportion of uninformative reports of $r=0.5$, and even strong beliefs near $0$ or $1$ will be skewed toward this focal point of $c=0.5$. This pattern of conservative behavior, which has been observed experimentally by, for example, Offerman et al. (2009) and in the experiment of this paper, is explained by the prospect theory model in Sect. 2 of this paper.^10^

The predictions of this theory reinforce the existing result that agents may not reveal their true beliefs even when assessed by a proper scoring rule, and provide an explanation for when and why we might expect to see these two forms of distortions. As demonstrated in Sect. 2, both effects appear to be largely driven by loss aversion, which motivates the agents to seek a certain payoff when they have moderate beliefs and to lower their risk by generally shading their reports closer toward $1/2$ for stronger beliefs. The intuition here is that reporting something other than $1/2$ introduces uncertainty into the payoffs, so that some outcomes will be felt as gains and some outcomes will be felt as losses. As a result, a loss-averse agent with beliefs close to $1/2$ (who doesn’t have much better information than the default baseline prediction) will not find it worthwhile to expose herself to the possibility of these losses. The * L*-adjusted QSR, which generalizes the classical QSR, can be calibrated to correct for both forms of distortions predicted by this prospect theory model of optimal reports. The $L$-adjusted QSR provides a simple scoring rule that can be used in a straightforward manner to elicit an agent’s true subjective probabilistic beliefs. The main challenge in successfully implementing this rule is that the optimal choice of $L^*$ requires an accurate estimate of the agent’s parameters $\alpha$, $\lambda$, and $w(p)$. In particular, when applying this adjustment the decision maker needs to be careful not to use an unsuitable value of $L$. For example, an agent who is truly risk neutral will respond to an $L$-adjusted scoring rule by biasing her reports away from $1/2$ for any choice of $L>1$.

Our experimental results demonstrate that the biases in people’s reports respond to the adjusted QSR as predicted by the theory. Our data suggests that the optimal calibration of $L^*=3.7$ for the average population does indeed perform better than the other treatments, but even the moderate-sized correction of $L=1.5$ provides a vast improvement over the classical unadjusted QSR. The major potential benefits of this $L$-adjustment include eliminating the flat region of reports $r=1/2$ for moderate beliefs, which are uninformative and prevent the optimal report function from being inverted, and de-biasing reports, so that they provide truthful subjective beliefs on average.

In theory, when processing reports, an decision maker would need to implement an additional second step of computing $r^{*-1}_L(\cdot )$ and inferring true beliefs according to $r^{*-1}_L(r)$ rather than simply using the raw report $r$ as the estimate. In practice, however, the impact of this additional step will be very small and likely dwarfed by noise in the reports and errors in the calibration of $L$ to the agent. Our experiment confirms that the second step is indeed unnecessary, and that reports can be simply recorded as provided in a straightforward manner.

For the general population, $L=3.7$ does seem to be the best adjustment to use, as predicted by applying existing empirical estimation of population parameters to our theoretical results and as evidenced in our experiment. Importantly, a more laborious procedure in which we provide each subject with an individually calibrated * L*-adjusted rule produces slightly worse results. The difference in performance is small though, and far from significant. One possible explanation is that we did not estimate subjects’ loss aversion parameters with sufficient precision. An avenue for future research is to try to improve the results of the IC treatment by estimating a subject’s loss-aversion parameter on the basis of a series of choices. Our conjecture is that the potential benefits of such an approach are limited. As the results of our paper indicate, no systematic risk bias remains when subjects are adjusted with the $L^*=3.7$ rule. Moreover, absolute differences between reported and true probabilities are small under this approach, leaving very little scope for improvement.

Finally, we would like to emphasize that while we only formally examined $L$-adjustments to a QSR, an exactly analogous adjustment could be applied to any other proper scoring rule with bounded payoffs. Applying the same analysis of behavior under risk will yield similar results; we would expect loss aversion to induce both a region of uninformative baseline reports for moderate beliefs and reports that are biased away from the agent’s true belief for stronger beliefs. The same $L$-adjustment should be equally effective at recovering informative responses by pulling the endpoints of the interval of baseline reports together until this “flat region” in the response function is eliminated. While there exists a closed-form solution for these results under the QSR, these optimal response functions and $L$-adjustments would have to be solved numerically for more general scoring rules.

### Electronic supplementary material

Below is the link to the electronic supplementary material.

Supplementary material 1 (doc 840 KB)

## Appendix 1

### Reporting under a general asymmetric * L*-adjusted QSR

To derive a closed-form solution for the optimal reporting strategy in response to a general asymmetric * L*-adjusted QSR, we need to make an additional assumption on reporting behavior:

#### **Definition 2**

(*Directional Reporting*) We say that the agent’s reporting preferences are **directional** if $p \le c \Rightarrow r \le c$ and $p \ge c \Rightarrow r \ge c$ for any beliefs $p$.

Directional reporting holds automatically in the optimal reporting strategy when the baseline $c=0.5$ (this follows through a symmetry argument, see the proof of Proposition 1 for details) as in the classical QSR, and approximately for a broad range of realistic parameter values when $c \ne 0.5$. Assuming directional reports is natural in the context of real-world agents who are asked to provide a report of their beliefs relative to some baseline, asserting that reporting behavior will be restricted to shading reports either toward or away from the baseline probability of $c$. While this assumption is not required to compute an agent’s optimal consistent report function, we will assume that it holds for the analysis that follows because it allows for closed-form solutions.

#### **Proposition 3**

*If the agent reports directionally, the optimal consistent report function to an asymmetric* *L*-*adjusted QSR is*

$$\begin{aligned} r_L^*(p) = \left\{ \begin{array}{ll} \frac{ \varLambda (p)^{{{\frac{1}{\alpha }}}} }{ \varLambda (p)^{{\frac{1}{\alpha }}} + L }, & p < \min \left\{ c, w_-^{-1} \left( \frac{\left( \frac{cL}{1-c}\right) ^{\alpha }}{\lambda +\left( \frac{cL}{1-c}\right) ^{\alpha }} \right) \right\} \\ c, & \min \left\{ c, w_-^{-1} \left( \frac{\left( \frac{cL}{1-c}\right) ^{\alpha }}{\lambda +\left( \frac{cL}{1-c}\right) ^{\alpha }} \right) \right\} \, \le \, p \, \le\, \max \left\{ c, w_+^{-1} \left( \frac{ \lambda }{ \left( \frac{(1-c)L}{c}\right) ^\alpha + \lambda } \right) \right\} \\ \frac{ L }{ \varLambda (1-p)^{{\frac{1}{\alpha }}} + L }, & p > \max \left\{ c, w_+^{-1} \left( \frac{ \lambda }{ \left( \frac{(1-c)L}{c}\right) ^\alpha + \lambda } \right) \right\} , \end{array}\right. \end{aligned}$$

*where* $\varLambda (p) = \frac{ \lambda w_-(p) }{w_+(1-p)}$ *is the agent’s loss-weighted odds ratio of event* $A$.

As before, the results are preserved under arbitrary positive linear rescaling of the payoffs. In other words, for any positive linear rescaling of the payoffs $\tilde{S}_L(r) \equiv a S_L(r) + b$, $a>0, b \in {\mathbb{R}}$, the optimal consistent report remains $\tilde{r}_L^*(p) = r_L^*(p)$ and the corresponding optimal expected outcome is simply rescaled according to $\tilde{E}^*(p) = a E^*(p) +b$.

The decision maker should then calibrate the * L*-adjusted QSR by selecting the value of $L^*$ that solves

$$\begin{aligned} w_-^{-1} \left( \frac{\left( \frac{cL^*}{1-c}\right) ^{\alpha }}{\lambda +\left( \frac{cL^*}{1-c}\right) ^{\alpha }} \right) = w_+^{-1} \left( \frac{ \lambda }{ \left( \frac{(1-c)L^*}{c}\right) ^\alpha + \lambda } \right) \end{aligned}$$

in order to eliminate the flat region of uninformative reports of $c$.

### Proofs of Propositions 1–3 and Corollary 1

#### *Proof of Proposition 1*

This result is a special case of Proposition 3, with $L =1$, $a=1$, and $b=0$. Note that directionality holds automatically if $c=0.5$. To prove this, it is sufficient to show that $\check{E}^*(p) \ge {\hat{E}}^*(p)$ for $p \le 0.5$ and ${\hat{E}}^*(p) \ge \check{E}^*(p)$ for $p \ge 0.5$ whenever the expectations $\check{E}^*(p)$ and ${\hat{E}}^*(p)$ are both consistent. Observe that when $c=0.5$, $\check{E}(r \; | \; p) = {\hat{E}}(1-r \; | \; 1-p),$ where $E(r \; | \; p)$ denotes a consistent expectation from reporting $r$ when the probability beliefs is $p$. Since $\check{r}^*(p) = 1 - \hat{r}^*(1-p)$, we have that $\check{E}(\check{r}^*(p) \; | \; p) = \check{E}(1 - \hat{r}^*(1-p) \; | \; p) = {\hat{E}}(\hat{r}^*(1-p) \; | \; 1 - p),$ or $\check{E}^*(p) = {\hat{E}}^*(1-p)$. In particular, this means that $\check{E}^*(1/2) = {\hat{E}}^*(1/2)$ and $\check{E}^*(p)$ and ${\hat{E}}^*(p)$ are symmetric around $p=0.5$. Then to prove directionality it suffices to show that $\check{E}^*(p)$ is decreasing in $p$. As shown in Proposition 3, $\check{E}'(r) < 0$. $\frac{d}{dp} \check{r}^*(p) = \frac{ L \left( \frac{d}{dp} \varLambda (p)^{{\frac{1}{\alpha }}} \right) }{ \left( \varLambda (p)^{{\frac{1}{\alpha }}} + L \right) ^2 }$, where $\varLambda (p)^{{\frac{1}{\alpha }}} \ge 0$ and $\frac{d}{dp} \varLambda (p)^{{\frac{1}{\alpha }}} = {{\frac{1}{\alpha }}} \varLambda (p)^{({{\frac{1}{\alpha }}}-1)} \frac{w_+(1-p) \lambda w_-'(p) - \lambda w_-(p) w_+'(1-p) }{( w_+(1-p) )^2} \ge 0$, so $\frac{d}{dp} \check{r}^*(p) \ge 0$. Then by the chain rule $\frac{d}{dp}\check{E}^*(p) \le 0$, which implies that $\check{E}^*(p)$ is decreasing in $p$, ${\hat{E}}^*(p)$ is increasing in $p$, and the agent will prefer to report $r \le 0.5$ for $p \le 0.5$ and $r \ge 0.5$ for $p \ge 0.5$.$\square$

#### *Proof of Proposition 2*

This result also follows from Proposition 3, by setting $L = 1$ and comparing the case where $a=1$, and $b=0$ to the case of general $a$ and $b$. The optimal consistent report function is the same in both cases, and after simplifying the expressions for the corresponding consistent expected outcome, we also have that $\tilde{E}^*(p) = a E^*(p) +b$ for all $p$.$\square$

#### *Proof of Proposition 3*

By Lemma 1 of Palley (2015), for any risky prospect that yields a payoff of $$y$$ with probability $$p$$ and $$z$$ with probability $$1-p$$, where $$y \le z$$, there exists a unique consistent expected outcome $$E \in [y,z]$$ such that $$V(E) = E$$, which means there will be a unique $$E$$ associated with any report $$r. $$ Consider an * L*-adjusted asymmetric QSR whose payoffs have been rescaled by an arbitrary positive linear transformation $$a S_L(X,r) + b$$, $$a>0, b \in {\mathbb{R}}$$. The agent must consider three separate cases when selecting the value of $$r$$ to report:$$\square $$

**Case 1 ** ($r<c$): $$S_L(r) = \left\{ \begin{array}{ll} a \frac{(1-c)^2-(1-r)^2}{c^2 L} +b < b & {\text{if}}\; A\;  {\text{ occurs}}, \\ a \frac{c^2-r^2}{c^2} +b > b & {\text{if}}\;{\bar{A}} \; {\text{occurs}}, \end{array}\right. $$ meaning that $a \frac{(1-c)^2-(1-r)^2}{c^2 L} +b \, < \, E \, <\, a \frac{c^2-r^2}{c^2} + b$ and

$$\begin{aligned} v(S_L(r),E) = \left\{ \begin{array}{ll} E - \lambda \left( E - \left( a \frac{(1-c)^2-(1-r)^2}{c^2 L}+b\right) \right) ^\alpha \quad {\text{if}}\;A \; {\text{occurs}} \\ E + \left( a\frac{c^2-r^2}{c^2}+b-E\right) ^\alpha \quad {\text{if}} \; {\bar{A}}\; {\text{occurs}}. \end{array}\right. \end{aligned}$$

Consistency requires that $w_-(p) \left( E - \lambda (E - a\frac{(1-c)^2-(1-r)^2}{c^2 L}-b)^\alpha \right) + w_+(1-p) \left( E + (a\frac{c^2-r^2}{c^2}+b-E)^\alpha \right) = E,$ so the consistent expectation for $r<c$ is $\check{E}(r) = a \frac{ \frac{c^2-r^2}{c^2} + \varLambda (p)^{{\frac{1}{\alpha }}} \left( \frac{(1-c)^2-(1-r)^2}{c^2 L} \right) }{ 1+ \varLambda (p)^{{\frac{1}{\alpha }}} } +b$, where $\varLambda (p) \equiv \frac{\lambda w_-(p)}{w_+(1-p)}.$ $\check{E}$ is a concave quadratic function of $r$, so its maximum occurs where $\check{E}'(r)= \frac{2a}{c^2 \left( 1+ \varLambda (p)^{{\frac{1}{\alpha }}} \right) } \left( \frac{1}{L} \varLambda (p)^{{\frac{1}{\alpha }}} (1-r) - r \right) =0,$ meaning that $\check{r}^*= \frac{ \varLambda (p)^{{\frac{1}{\alpha }}} }{ \varLambda (p)^{{\frac{1}{\alpha }}} + L }.$ However, this is only consistent for $r<c$, so this reporting strategy is optimal only if $\frac{ \varLambda (p)^{{\frac{1}{\alpha }}} }{ \varLambda (p)^{{\frac{1}{\alpha }}} + L } < c$, or equivalently, only if $p < w_-^{-1} \left( \frac{(\frac{c L}{1-c})^{\alpha }}{\lambda +(\frac{c L}{1-c})^{\alpha }} \right) .$ Then for all $p < w_-^{-1} \left( \frac{(\frac{c L}{1-c})^{\alpha }}{\lambda +(\frac{c L}{1-c})^{\alpha }} \right)$, $\check{E}^*(p) = \check{E}\left({\frac{ \varLambda (p)^{{\frac{1}{\alpha }}} }{ \varLambda (p)^{{\frac{1}{\alpha }}} + L }}\right) > \check{E}(c)=b,$ and for all $p \ge w_-^{-1} \left( \frac{(\frac{c L}{1-c})^{\alpha }}{\lambda +(\frac{c L}{1-c})^{\alpha }} \right)$, reporting $r<c$ is not optimal.

**Case 2 **($r>c$): $$S_L(r) = \left\{ \begin{array}{l} a \frac{(1-c)^2-(1-r)^2}{(1-c)^2} +b < b \;\; {\text{if}}\; A\; {\text{occurs}} \\ a \frac{c^2-r^2}{(1-c)^2 L} +b > b \;\; {\text{if}}\; {\bar{A}} \;{\text{occurs,}} \end{array}\right. $$ meaning that $a \frac{c^2-r^2}{(1-c)^2 L} +b \, <\,  E \, < \, a \frac{(1-c)^2-(1-r)^2}{(1-c)^2} + b$ and

$$\begin{aligned} v(S_L(r),E) = \left\{ \begin{array}{l} E + \left( a \frac{(1-c)^2-(1-r)^2}{(1-c)^2}+b-E\right) ^\alpha \;\; {\text{if}}\; A\; {\text{occurs}} \\ E - \lambda (E - \left( a \frac{c^2-r^2}{(1-c)^2 L}+b \right) )^\alpha \;\; {\text {if}}\;{\bar{A}} \;{\text{occurs.}} \end{array}\right. \end{aligned}$$

Consistency requires that $w_+(p) \left( E + (a \frac{(1-c)^2-(1-r)^2}{(1-c)^2}+b -E)^\alpha \right) + w_-(1-p) \left( E - \lambda (E - a \frac{c^2-r^2}{(1-c)^2 L} -b)^\alpha \right) = E,$ so the consistent expectation for $r>c$ is ${\hat{E}}(r) =a \frac{ \frac{ (1-c)^2 -(1-r)^2}{(1-c)^2} + \varLambda (1-p)^{{\frac{1}{\alpha }}} \left( \frac{c^2-r^2}{(1-c)^2 L} \right) }{1+ \varLambda (1-p)^{{\frac{1}{\alpha }}} } +b.$ ${\hat{E}}$ is a concave quadratic function of $r$, so its maximum occurs where ${\hat{E}}'(r)= \frac{2a}{ \left( 1+ \varLambda (1-p)^{{\frac{1}{\alpha }}} \right) (1-c)^2} \left( (1-r) - \varLambda (1-p)^{{\frac{1}{\alpha }}} \frac{r}{L} \right) =0,$ meaning that $\hat{r}^*= \frac{ L }{ \varLambda (1-p)^{{\frac{1}{\alpha }}} +L }.$ However, this is only consistent for $r>c$, so this reporting strategy is optimal only if $\frac{ L }{ \varLambda (1-p)^{{\frac{1}{\alpha }}} +L } > c$, or equivalently, only if $p > w_+^{-1} \left( \frac{ \lambda }{ (\frac{(1-c)L}{c})^\alpha + \lambda } \right) .$ Then for all $p > w_+^{-1} \left( \frac{ \lambda }{ (\frac{(1-c)L}{c})^\alpha + \lambda } \right)$, ${\hat{E}}^*(p) = {\hat{E}}( \frac{ L }{ \varLambda (1-p)^{{\frac{1}{\alpha }}} +L } ) > {\hat{E}}(c)=b,$ and for all $p \le w_+^{-1} \left( \frac{ \lambda }{ (\frac{(1-c)L}{c})^\alpha + \lambda } \right)$, reporting $r>c$ is not optimal.

**Case 3 **($r=c$): $S(r) = b$, meaning that $E = b$ and $v(S(r),E) = b$. Then consistency is satisfied since $V(E) = b = E$.

Then for any belief $p$, the agent has three reporting choices:

1. $r<c$, in which case she will receive $\check{E}(\check{r}^*(p))$
2. $r>c$, in which case she will receive ${\hat{E}}(\hat{r}^*(p))$, or
3. $r=c$, in which case she will receive $E=b$.

If $w_-^{-1} \left( \frac{(\frac{c L}{1-c})^{\alpha }}{\lambda +(\frac{c L}{1-c})^{\alpha }} \right) < w_+^{-1} \left( \frac{ \lambda }{ (\frac{(1-c)L}{c})^\alpha + \lambda } \right)$, then the only consistent report for $p \in [w_-^{-1} \left( \frac{(\frac{c L}{1-c})^{\alpha }}{\lambda +(\frac{c L}{1-c})^{\alpha }} \right) , w_+^{-1} \left( \frac{ \lambda }{ (\frac{(1-c)L}{c})^\alpha + \lambda } \right) ]$ is $r = c$, $r^*= \frac{ \varLambda (p)^{\frac{1}{\alpha }} }{ \varLambda (p)^{\frac{1}{\alpha }} + L }$ for $p < w_-^{-1} \left( \frac{(\frac{c L}{1-c})^{\alpha }}{\lambda +(\frac{c L}{1-c})^{\alpha }} \right)$, and $r^*= \frac{ L }{ \varLambda (1-p)^{\frac{1}{\alpha }} +L }$ for $p > w_+^{-1} \left( \frac{ \lambda }{ (\frac{(1-c)L}{c})^\alpha + \lambda } \right)$.

If $w_-^{-1} \left( \frac{(\frac{c L}{1-c})^{\alpha }}{\lambda +(\frac{c L}{1-c})^{\alpha }} \right) \ge w_+^{-1} \left( \frac{ \lambda }{ (\frac{(1-c)L}{c})^\alpha + \lambda } \right)$, then for $p < w_+^{-1} \left( \frac{ \lambda }{ (\frac{(1-c)L}{c})^\alpha + \lambda } \right)$ we have $r^*= \frac{ \varLambda (p)^{\frac{1}{\alpha }} }{ \varLambda (p)^{\frac{1}{\alpha }} + L }$, for $p > w_-^{-1} \left( \frac{(\frac{c L}{1-c})^{\alpha }}{\lambda +(\frac{c L}{1-c})^{\alpha }} \right)$ we have $r^*= \frac{ L }{ \varLambda (1-p)^{\frac{1}{\alpha }} +L }$, and for $p \in [ w_+^{-1} \left( \frac{ \lambda }{ (\frac{(1-c)L}{c})^\alpha + \lambda } \right) , w_-^{-1} \left( \frac{(\frac{c L}{1-c})^{\alpha }}{\lambda +(\frac{c L}{1-c})^{\alpha }} \right) ]$ we have $r^* = \arg \max \left\{ \check{E}({\frac{ \varLambda (p)^{\frac{1}{\alpha }} }{ \varLambda (p)^{\frac{1}{\alpha }} + L }}), {\hat{E}}( \frac{ L }{ \varLambda (1-p)^{\frac{1}{\alpha }} +L } ) \right\}$. Under the assumption of directional reporting, this simply reduces to $r^* = \frac{ L }{ \varLambda (1-p)^{\frac{1}{\alpha }} +L }$ for $p \in [w_-^{-1} \left( \frac{(\frac{c L}{1-c})^{\alpha }}{\lambda +(\frac{c L}{1-c})^{\alpha }} \right) ,c]$ and $r^* = \frac{ \varLambda (p)^{\frac{1}{\alpha }} }{ \varLambda (p)^{\frac{1}{\alpha }} + L }$ for $p \in [ c, w_+^{-1} \left( \frac{ \lambda }{ (\frac{(1-c)L}{c})^\alpha + \lambda } \right) ]$.

The consistent *ex ante* expected outcome corresponding to the optimal consistent report $r^*(p)$ is $E^*(p) =$

$$\begin{aligned} \left\{ \begin{array}{ll} a \frac{ c^2-\left( \frac{ \varLambda (p)^{\frac{1}{\alpha }} }{ \varLambda (p)^{\frac{1}{\alpha }} + L } \right) ^2 + \frac{\varLambda (p)^{\frac{1}{\alpha }}}{L} \left( (1-c)^2- \left( \frac{ L }{ \varLambda (p)^{\frac{1}{\alpha }} + L } \right) ^2 \right) }{ c^2 \left( 1+ \varLambda (p)^{\frac{1}{\alpha }} \right) } +b , \quad p < \min \left\{ c,\right. \\ \left. w_-^{-1} \left( \frac{\left( \frac{cL}{1-c}\right) ^{\alpha }}{\lambda +\left( \frac{cL}{1-c}\right) ^{\alpha }} \right) \right\} , \\ b, \quad\min \left\{ c, w_-^{-1} \left( \frac{(\frac{cL}{1-c})^{\alpha }}{\lambda +\left( \frac{cL}{1-c}\right) ^{\alpha }} \right) , \right\} \le p \le \max \left\{ c, w_+^{-1} \left( \frac{ \lambda }{ \left( \frac{(1-c)L}{c}\right) ^\alpha + \lambda } \right) \right\} , \\ a \frac{ (1-c)^2 - \left( \frac{ \varLambda (1-p)^{\frac{1}{\alpha }} }{ \varLambda (1-p)^{\frac{1}{\alpha }} +L } \right) ^2 + \frac{ \varLambda (1-p)^{\frac{1}{\alpha }}}{L} \left( c^2 - \left( \frac{ L }{ \varLambda (1-p)^{\frac{1}{\alpha }} +L } \right) ^2 \right) }{(1-c)^2 \left( 1+ \varLambda (1-p)^{\frac{1}{\alpha }} \right) } +b , \quad p > \max \left\{ c, \right.  \left. w_+^{-1} \left( \frac{ \lambda }{ \left( \frac{(1-c)L}{c}\right) ^\alpha + \lambda } \right) \right\} . \end{array}\right. \end{aligned}$$

#### *Proof of Corollary 1*

If $c=1/2$, then we need $L^*$ to satisfy $w_-^{-1} \left( \frac{ (L^*)^{\alpha } }{\lambda +(L^*)^{\alpha }} \right) = w_+^{-1} \left( \frac{ \lambda }{ (L^*)^{\alpha } + \lambda } \right)$, or $w_-^{-1} \left( \frac{ (L^*)^{\alpha } }{\lambda +(L^*)^{\alpha }} \right) = 1 - w_-^{-1} \left( \frac{ (L^*)^{\alpha } }{ (L^*)^{\alpha } + \lambda } \right)$. This means that $1/2 = w_-^{-1} \left( \frac{ (L^*)^{\alpha } }{ (L^*)^{\alpha } + \lambda } \right)$, or $(L^*)^{\alpha } w_-(1/2) + \lambda w_-(1/2) = (L^*)^{\alpha }$. Rearranging terms, $(L^*)^{\alpha } = \frac{\lambda w_-(1/2)}{1-w_-(1/2)},$ which yields the desired result.$\square$

## Footnotes

1 References include McKelvey and Page (1990), Offerman et al. (1996), Huck and Weizsäcker (2002), Nyarko and Schotter (2002), Costa-Gomes and Weizsäcker (2008), Armantier and Treich (2013), Offerman et al. (2009), Blanco et al. (2010), Andersen et al. (2010), and Kothiyal et al. (2011). For a survey of the work on scoring rules and other belief elicitation methods, see Schlag et al. (2014)

2 The decision maker may find it useful to select an asymmetric baseline $c \ne 1/2$ if he expects the assessed probability of the event to be particularly low (e.g. the probability of rain on a given day in a desert location) or high (e.g. the probability of rain on a given day in a rainforest location). However, in practice this baseline is usually taken to be $c=1/2$, as in the classical QSR.

3 Several existing studies estimate average parameters $\lambda$ and $\alpha$ for the general population using the classical cumulative prospect theory model of Tversky and Kahneman (1992), who find $\lambda = 2.25$ and $\alpha = 0.88$. In recent work, Tu (2005) finds $\lambda = 3.18$ and $\alpha = 0.68$, Abdellaoui et al. (2007) find $\lambda = 2.54$ and $\alpha = 0.72$, and Booij et al. (2010) estimate $\lambda = 1.58$ and $\alpha = 0.86$. While these estimates are derived from a different model of risk (cumulative prospect theory with a fixed rather than endogenous reference point), their interpretation corresponds directly to our model, so we use an average of these estimates as a rough benchmark for a representative agent of the general population.

4 These assumptions hold trivially for the unweighted case $w(p)=p$ and approximately for most existing estimates of weighting functions that overweight low probabilities and underweight high probabilities. For example, using Goldstein and Einhorn’s (1987) parameterization $w(p) = \frac{\delta p^\gamma }{\delta p^\gamma + (1-p)^\gamma }$, Abdellaoui (2000) finds $\delta _+ = 0.65$, $\gamma _+ = 0.60$, $\delta _- = 0.84$ and $\gamma _- = 0.65$, Abdellaoui et al. (2005) find $\delta _+ = 0.98$, $\gamma _+ = 0.83$, $\delta _- = 1.35$ and $\gamma _- = 0.84$ and Booij et al. (2010) estimate $\delta _+ = 0.77$, $\gamma _+ = 0.62$, $\delta _- = 1.02$ and $\gamma _- = 0.59$. We use a rough average of these existing estimates as a benchmark for a representative agent for the overall population, but a number of other functional forms for the weighting functions could be used as well (see, e.g., Prelec (1998)).

5 Further details about the mechanics and intuition of this model can be found in Palley (2015). In this case decision weights reduce simply to weighted probabilities because there are only two possible outcomes.

6 If $\alpha = 1$, $\lambda = 1$, and $w(p) = p$, then the optimal report is $r^*(p) = p$ and $E^*(p) = {\mathbb{E}}[S(X,p)]$, the expected score function in the simpler risk-neutral model (see Winkler (1994)). This behavioral model therefore includes the risk-neutral model that proper scoring rules are based on as a special case, yielding a consistent prediction regarding the reports and expected outcome. If the agent is risk-neutral, does not over- or under-weight probabilities, and does not exhibit loss aversion, then the QSR retains its ex ante incentives for truthful reporting.

7 More generally, for any two outcomes $x_1 \le x_2$, where $x_1$ occurs with probability $p$ and $x_2$ occurs with probability $1-p$, the consistent expectation in this case can be written as $E = ( x_2 + \lambda p x_1 / (1-p) ) / ( 1 + \lambda p / (1-p) )$.

8 We are grateful to an anonymous referee for suggesting this treatment.

9 In total, 6 subjects in NC, 2 subjects in MC and 1 subject in IC reported 50 % in every round, while no subject in the LC treatment reported 50 % in every round. In contrast, the proportion of consistent reporters with a coefficient of at least 0.9 equaled 60.0 % in NC, 73.8 % in MC, 78.0 % in IC and 80.4 % in LC.

10 In situations where agents receive rewards from reporting beliefs and from making additional decisions, other distortions may emerge. In such cases, agents may hedge their beliefs, for example in order to guarantee a minimal payoff. The extent to which hedging biases reports when beliefs are incentivized is discussed in Blanco et al. (2010) and Armantier and Treich (2013).
