# Supplementary material for: Lossed in translation: an off-the-shelf method to recover probabilistic beliefs from loss-averse agents
Source: Exp Econ. 2015 Apr 17;19(1):1–30. doi: 10.1007/s10683-015-9429-0 (PMC5153699; doi:10.1007/s10683-015-9429-0)
Supplement: Supplementary file 1 — Supplementary material 1 (doc 840 KB) [file 10683_2015_9429_MOESM1_ESM.doc]

**Appendix B: Experimental Instructions and Interface**

Welcome to this experiment on decision-making. Please read the following instructions carefully. As soon as everyone has finished reading the instructions you will receive a handout with a summary. During the experiment you will be asked to make a number of decisions. Your decisions will determine your earnings. (In this experiment, your earnings are not affected by the decisions of other participants). The experiment consists of 20 rounds. At the end of the experiment, one of the 20 rounds is selected at random. Your earnings for the experiment will equal the earnings that you made in this round. Your earnings will be privately paid to you in cash.

THE TASK

In this experiment, you will be asked to give probability judgments. In each round, you will be asked to give your probability judgment that a randomly drawn number will be in a particular range. The randomly drawn number will always be an integer number between 0 and 100, and each of the possible numbers between 0 and 100 will be equally likely. The range will differ across rounds. In the round that is selected for payment, your earnings will be determined as follows.

1. The computer draws a number between 0 and 100 (each number is equally likely).

2. Then it will be determined whether the number is in the range for the particular round or not.

3. You will receive a payoff that depends on your probability judgment for that round and on whether the number was in the range or not.

PAYOFF

On your table you find the list of payoffs that will result for each possible probability judgment, both for the case that the number is in the range and the case that it is outside of the range.

EXAMPLE

(The numbers in this example are arbitrarily chosen and do not indicate how you should make your choices.)

Assume that in the round that is selected for payment you were asked to give your probability judgment that the number will be in the range of 0-27. Say that you chose a probability judgment of 35%. If in that case the randomly drawn number was in the range you will receive a payoff of 9.93 euro and if it was outside of the range you will receive a payoff of 13.53 euro. The borders of the range belong to the range. For instance, if for the round of the example a number of 0 or 27 is drawn, then the number is in the range.

MAKING YOUR DECISIONS

The input of your probability judgment takes place in two phases: first you type in an integer number between 0 and 100, next you will be shown a menu in which your choice is replicated with the corresponding scores from the table. At that moment you can still alter your choice and choose any other integer number between 0 and 100. You can do this by selecting the up or down arrow, or by clicking the mouse in the menu and scroll to another probability judgment. Next, when you click on “Satisfied with choice” your choice is final and you continue with the next round. Below you see an example of the decision-making screen that will be used in the experiment. At this moment, you cannot yet make a decision.


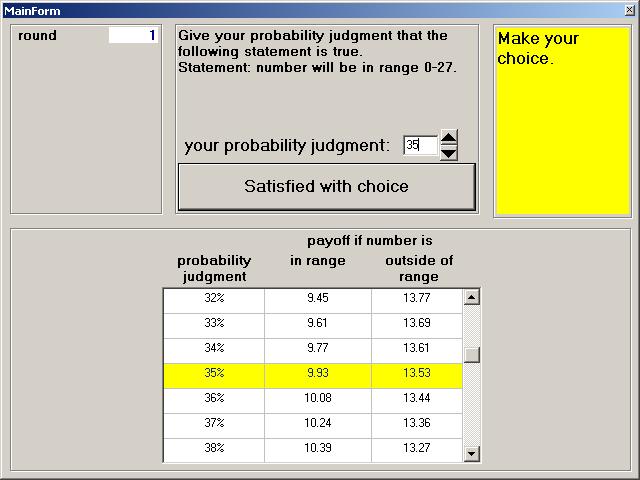


In this experiment there are no right or wrong answers; you choose what you want best. On the next screen you will be asked to answer some control questions. Please answer these questions now.

Please answer the following questions:

| (1) Is the following statement correct? In this experiment, your earnings will be the sum of what you earn in all rounds. | yes | no |
| --- | --- | --- |
|  |  |  |
| (2) Is the following statement correct? In the round that is selected for payment, the computer will draw an integer number between 0 and 100, and each of these numbers is equally likely. | yes | no |
|  |  |  |
|  |  |  |
| (3) THE FOLLOWING DECISIONS ARE IMAGINARY AND DO NOT INDICATE WHAT YOU SHOULD DO IN THE EXPERIMENT. Consider that a round is selected for actual payment in which the range of numbers equals [0-70]. You chose a probability judgment of 55% |  |  |
| (A) How much will you earn (in eurocents) if the number randomly drawn by the computer is 64? |  |  |
| (B) How much will you earn (in eurocents) if the number randomly drawn by the computer is 92? |  |  |
| (4) THE FOLLOWING DECISIONS ARE IMAGINARY AND DO NOT INDICATE WHAT YOU SHOULD DO IN THE EXPERIMENT. Consider that a round is selected for actual payment in which the range of numbers equals [0-70]. You chose a probability judgment of 82% |  |  |
| (A) How much will you earn (in eurocents) if the number randomly drawn by the computer is 15? |  |  |
| (B) How much will you earn (in eurocents) if the number randomly drawn by the computer is 77? |  |  |

**Payoff Table: Treatment NC**

|  | **Payoff (in euros)**  **if number is** | |  |  | **Payoff (in euros)**  **if number** | |
| --- | --- | --- | --- | --- | --- | --- |
| **Probability judgment** | **In range** | **Outside of range** |  | **Probability judgment** | **In range** | **Outside of range** |
| 0% | 3.00 | 15.00 |  | 51% | 12.12 | 11.88 |
| 1% | 3.24 | 15.00 |  | 52% | 12.24 | 11.76 |
| 2% | 3.48 | 15.00 |  | 53% | 12.35 | 11.63 |
| 3% | 3.71 | 14.99 |  | 54% | 12.46 | 11.50 |
| 4% | 3.94 | 14.98 |  | 55% | 12.57 | 11.37 |
| 5% | 4.17 | 14.97 |  | 56% | 12.68 | 11.24 |
| 6% | 4.40 | 14.96 |  | 57% | 12.78 | 11.10 |
| 7% | 4.62 | 14.94 |  | 58% | 12.88 | 10.96 |
| 8% | 4.84 | 14.92 |  | 59% | 12.98 | 10.82 |
| 9% | 5.06 | 14.90 |  | 60% | 13.08 | 10.68 |
| 10% | 5.28 | 14.88 |  | 61% | 13.17 | 10.53 |
| 11% | 5.49 | 14.85 |  | 62% | 13.27 | 10.39 |
| 12% | 5.71 | 14.83 |  | 63% | 13.36 | 10.24 |
| 13% | 5.92 | 14.80 |  | 64% | 13.44 | 10.08 |
| 14% | 6.12 | 14.76 |  | 65% | 13.53 | 9.93 |
| 15% | 6.33 | 14.73 |  | 66% | 13.61 | 9.77 |
| 16% | 6.53 | 14.69 |  | 67% | 13.69 | 9.61 |
| 17% | 6.73 | 14.65 |  | 68% | 13.77 | 9.45 |
| 18% | 6.93 | 14.61 |  | 69% | 13.85 | 9.29 |
| 19% | 7.13 | 14.57 |  | 70% | 13.92 | 9.12 |
| 20% | 7.32 | 14.52 |  | 71% | 13.99 | 8.95 |
| 21% | 7.51 | 14.47 |  | 72% | 14.06 | 8.78 |
| 22% | 7.70 | 14.42 |  | 73% | 14.13 | 8.61 |
| 23% | 7.89 | 14.37 |  | 74% | 14.19 | 8.43 |
| 24% | 8.07 | 14.31 |  | 75% | 14.25 | 8.25 |
| 25% | 8.25 | 14.25 |  | 76% | 14.31 | 8.07 |
| 26% | 8.43 | 14.19 |  | 77% | 14.37 | 7.89 |
| 27% | 8.61 | 14.13 |  | 78% | 14.42 | 7.70 |
| 28% | 8.78 | 14.06 |  | 79% | 14.47 | 7.51 |
| 29% | 8.95 | 13.99 |  | 80% | 14.52 | 7.32 |
| 30% | 9.12 | 13.92 |  | 81% | 14.57 | 7.13 |
| 31% | 9.29 | 13.85 |  | 82% | 14.61 | 6.93 |
| 32% | 9.45 | 13.77 |  | 83% | 14.65 | 6.73 |
| 33% | 9.61 | 13.69 |  | 84% | 14.69 | 6.53 |
| 34% | 9.77 | 13.61 |  | 85% | 14.73 | 6.33 |
| 35% | 9.93 | 13.53 |  | 86% | 14.76 | 6.12 |
| 36% | 10.08 | 13.44 |  | 87% | 14.80 | 5.92 |
| 37% | 10.24 | 13.36 |  | 88% | 14.83 | 5.71 |
| 38% | 10.39 | 13.27 |  | 89% | 14.85 | 5.49 |
| 39% | 10.53 | 13.17 |  | 90% | 14.88 | 5.28 |
| 40% | 10.68 | 13.08 |  | 91% | 14.90 | 5.06 |
| 41% | 10.82 | 12.98 |  | 92% | 14.92 | 4.84 |
| 42% | 10.96 | 12.88 |  | 93% | 14.94 | 4.62 |
| 43% | 11.10 | 12.78 |  | 94% | 14.96 | 4.40 |
| 44% | 11.24 | 12.68 |  | 95% | 14.97 | 4.17 |
| 45% | 11.37 | 12.57 |  | 96% | 14.98 | 3.94 |
| 46% | 11.50 | 12.46 |  | 97% | 14.99 | 3.71 |
| 47% | 11.63 | 12.35 |  | 98% | 15.00 | 3.48 |
| 48% | 11.76 | 12.24 |  | 99% | 15.00 | 3.24 |
| 49% | 11.88 | 12.12 |  | 100% | 15.00 | 3.00 |
| 50% | 12.00 | 12.00 |  |  |  |  |

**Payoff Table: Treatment MC**

|  | **Payoff (in euros)**  **if number is** | |  |  | **Payoff (in euros)**  **if number** | |
| --- | --- | --- | --- | --- | --- | --- |
| **Probability judgment** | **In range** | **Outside of range** |  | **Probability judgment** | **In range** | **Outside of range** |
| 0% | 6.00 | 15.00 |  | 51% | 12.12 | 11.92 |
| 1% | 6.16 | 15.00 |  | 52% | 12.24 | 11.84 |
| 2% | 6.32 | 15.00 |  | 53% | 12.35 | 11.75 |
| 3% | 6.47 | 14.99 |  | 54% | 12.46 | 11.67 |
| 4% | 6.63 | 14.98 |  | 55% | 12.57 | 11.58 |
| 5% | 6.78 | 14.97 |  | 56% | 12.68 | 11.49 |
| 6% | 6.93 | 14.96 |  | 57% | 12.78 | 11.40 |
| 7% | 7.08 | 14.94 |  | 58% | 12.88 | 11.31 |
| 8% | 7.23 | 14.92 |  | 59% | 12.98 | 11.22 |
| 9% | 7.38 | 14.90 |  | 60% | 13.08 | 11.12 |
| 10% | 7.52 | 14.88 |  | 61% | 13.17 | 11.02 |
| 11% | 7.66 | 14.85 |  | 62% | 13.27 | 10.92 |
| 12% | 7.80 | 14.83 |  | 63% | 13.36 | 10.82 |
| 13% | 7.94 | 14.80 |  | 64% | 13.44 | 10.72 |
| 14% | 8.08 | 14.76 |  | 65% | 13.53 | 10.62 |
| 15% | 8.22 | 14.73 |  | 66% | 13.61 | 10.52 |
| 16% | 8.36 | 14.69 |  | 67% | 13.69 | 10.41 |
| 17% | 8.49 | 14.65 |  | 68% | 13.77 | 10.30 |
| 18% | 8.62 | 14.61 |  | 69% | 13.85 | 10.19 |
| 19% | 8.75 | 14.57 |  | 70% | 13.92 | 10.08 |
| 20% | 8.88 | 14.52 |  | 71% | 13.99 | 9.97 |
| 21% | 9.01 | 14.47 |  | 72% | 14.06 | 9.85 |
| 22% | 9.13 | 14.42 |  | 73% | 14.13 | 9.74 |
| 23% | 9.26 | 14.37 |  | 74% | 14.19 | 9.62 |
| 24% | 9.38 | 14.31 |  | 75% | 14.25 | 9.50 |
| 25% | 9.50 | 14.25 |  | 76% | 14.31 | 9.38 |
| 26% | 9.62 | 14.19 |  | 77% | 14.37 | 9.26 |
| 27% | 9.74 | 14.13 |  | 78% | 14.42 | 9.13 |
| 28% | 9.85 | 14.06 |  | 79% | 14.47 | 9.01 |
| 29% | 9.97 | 13.99 |  | 80% | 14.52 | 8.88 |
| 30% | 10.08 | 13.92 |  | 81% | 14.57 | 8.75 |
| 31% | 10.19 | 13.85 |  | 82% | 14.61 | 8.62 |
| 32% | 10.30 | 13.77 |  | 83% | 14.65 | 8.49 |
| 33% | 10.41 | 13.69 |  | 84% | 14.69 | 8.36 |
| 34% | 10.52 | 13.61 |  | 85% | 14.73 | 8.22 |
| 35% | 10.62 | 13.53 |  | 86% | 14.76 | 8.08 |
| 36% | 10.72 | 13.44 |  | 87% | 14.80 | 7.94 |
| 37% | 10.82 | 13.36 |  | 88% | 14.83 | 7.80 |
| 38% | 10.92 | 13.27 |  | 89% | 14.85 | 7.66 |
| 39% | 11.02 | 13.17 |  | 90% | 14.88 | 7.52 |
| 40% | 11.12 | 13.08 |  | 91% | 14.90 | 7.38 |
| 41% | 11.22 | 12.98 |  | 92% | 14.92 | 7.23 |
| 42% | 11.31 | 12.88 |  | 93% | 14.94 | 7.08 |
| 43% | 11.40 | 12.78 |  | 94% | 14.96 | 6.93 |
| 44% | 11.49 | 12.68 |  | 95% | 14.97 | 6.78 |
| 45% | 11.58 | 12.57 |  | 96% | 14.98 | 6.63 |
| 46% | 11.67 | 12.46 |  | 97% | 14.99 | 6.47 |
| 47% | 11.75 | 12.35 |  | 98% | 15.00 | 6.32 |
| 48% | 11.84 | 12.24 |  | 99% | 15.00 | 6.16 |
| 49% | 11.92 | 12.12 |  | 100% | 15.00 | 6.00 |
| 50% | 12.00 | 12.00 |  |  |  |  |

**Payoff Table: Treatment LC**

|  | **Payoff (in euros)**  **if number is** | |  |  | **Payoff (in euros)**  **if number** | |
| --- | --- | --- | --- | --- | --- | --- |
| **Probability judgment** | **In range** | **Outside of range** |  | **Probability judgment** | **In range** | **Outside of range** |
| 0% | 9.57 | 15.00 |  | 51% | 12.12 | 11.97 |
| 1% | 9.63 | 15.00 |  | 52% | 12.24 | 11.93 |
| 2% | 9.70 | 15.00 |  | 53% | 12.35 | 11.90 |
| 3% | 9.76 | 14.99 |  | 54% | 12.46 | 11.87 |
| 4% | 9.82 | 14.98 |  | 55% | 12.57 | 11.83 |
| 5% | 9.88 | 14.97 |  | 56% | 12.68 | 11.79 |
| 6% | 9.95 | 14.96 |  | 57% | 12.78 | 11.76 |
| 7% | 10.01 | 14.94 |  | 58% | 12.88 | 11.72 |
| 8% | 10.07 | 14.92 |  | 59% | 12.98 | 11.68 |
| 9% | 10.13 | 14.90 |  | 60% | 13.08 | 11.64 |
| 10% | 10.18 | 14.88 |  | 61% | 13.17 | 11.60 |
| 11% | 10.24 | 14.85 |  | 62% | 13.27 | 11.56 |
| 12% | 10.30 | 14.83 |  | 63% | 13.36 | 11.52 |
| 13% | 10.36 | 14.80 |  | 64% | 13.44 | 11.48 |
| 14% | 10.41 | 14.76 |  | 65% | 13.53 | 11.44 |
| 15% | 10.47 | 14.73 |  | 66% | 13.61 | 11.40 |
| 16% | 10.52 | 14.69 |  | 67% | 13.69 | 11.35 |
| 17% | 10.58 | 14.65 |  | 68% | 13.77 | 11.31 |
| 18% | 10.63 | 14.61 |  | 69% | 13.85 | 11.27 |
| 19% | 10.68 | 14.57 |  | 70% | 13.92 | 11.22 |
| 20% | 10.74 | 14.52 |  | 71% | 13.99 | 11.18 |
| 21% | 10.79 | 14.47 |  | 72% | 14.06 | 11.13 |
| 22% | 10.84 | 14.42 |  | 73% | 14.13 | 11.08 |
| 23% | 10.89 | 14.37 |  | 74% | 14.19 | 11.03 |
| 24% | 10.94 | 14.31 |  | 75% | 14.25 | 10.99 |
| 25% | 10.99 | 14.25 |  | 76% | 14.31 | 10.94 |
| 26% | 11.03 | 14.19 |  | 77% | 14.37 | 10.89 |
| 27% | 11.08 | 14.13 |  | 78% | 14.42 | 10.84 |
| 28% | 11.13 | 14.06 |  | 79% | 14.47 | 10.79 |
| 29% | 11.18 | 13.99 |  | 80% | 14.52 | 10.74 |
| 30% | 11.22 | 13.92 |  | 81% | 14.57 | 10.68 |
| 31% | 11.27 | 13.85 |  | 82% | 14.61 | 10.63 |
| 32% | 11.31 | 13.77 |  | 83% | 14.65 | 10.58 |
| 33% | 11.35 | 13.69 |  | 84% | 14.69 | 10.52 |
| 34% | 11.40 | 13.61 |  | 85% | 14.73 | 10.47 |
| 35% | 11.44 | 13.53 |  | 86% | 14.76 | 10.41 |
| 36% | 11.48 | 13.44 |  | 87% | 14.80 | 10.36 |
| 37% | 11.52 | 13.36 |  | 88% | 14.83 | 10.30 |
| 38% | 11.56 | 13.27 |  | 89% | 14.85 | 10.24 |
| 39% | 11.60 | 13.17 |  | 90% | 14.88 | 10.18 |
| 40% | 11.64 | 13.08 |  | 91% | 14.90 | 10.13 |
| 41% | 11.68 | 12.98 |  | 92% | 14.92 | 10.07 |
| 42% | 11.72 | 12.88 |  | 93% | 14.94 | 10.01 |
| 43% | 11.76 | 12.78 |  | 94% | 14.96 | 9.95 |
| 44% | 11.79 | 12.68 |  | 95% | 14.97 | 9.88 |
| 45% | 11.83 | 12.57 |  | 96% | 14.98 | 9.82 |
| 46% | 11.87 | 12.46 |  | 97% | 14.99 | 9.76 |
| 47% | 11.90 | 12.35 |  | 98% | 15.00 | 9.70 |
| 48% | 11.93 | 12.24 |  | 99% | 15.00 | 9.63 |
| 49% | 11.97 | 12.12 |  | 100% | 15.00 | 9.57 |
| 50% | 12.00 | 12.00 |  |  |  |  |

**Payoff Tables: Treatment IC**

**Option 1 (L=1)**

|  | **Payoff (in euros)**  **if number is** | |  |  | **Payoff (in euros)**  **if number** | |
| --- | --- | --- | --- | --- | --- | --- |
| **Probability judgment** | **In range** | **Outside of range** |  | **Probability judgment** | **In range** | **Outside of range** |
| 0% | 3.00 | 15.00 |  | 51% | 12.12 | 11.88 |
| 1% | 3.24 | 15.00 |  | 52% | 12.24 | 11.76 |
| 2% | 3.48 | 15.00 |  | 53% | 12.35 | 11.63 |
| 3% | 3.71 | 14.99 |  | 54% | 12.46 | 11.50 |
| 4% | 3.94 | 14.98 |  | 55% | 12.57 | 11.37 |
| 5% | 4.17 | 14.97 |  | 56% | 12.68 | 11.24 |
| 6% | 4.40 | 14.96 |  | 57% | 12.78 | 11.10 |
| 7% | 4.62 | 14.94 |  | 58% | 12.88 | 10.96 |
| 8% | 4.84 | 14.92 |  | 59% | 12.98 | 10.82 |
| 9% | 5.06 | 14.90 |  | 60% | 13.08 | 10.68 |
| 10% | 5.28 | 14.88 |  | 61% | 13.17 | 10.53 |
| 11% | 5.49 | 14.85 |  | 62% | 13.27 | 10.39 |
| 12% | 5.71 | 14.83 |  | 63% | 13.36 | 10.24 |
| 13% | 5.92 | 14.80 |  | 64% | 13.44 | 10.08 |
| 14% | 6.12 | 14.76 |  | 65% | 13.53 | 9.93 |
| 15% | 6.33 | 14.73 |  | 66% | 13.61 | 9.77 |
| 16% | 6.53 | 14.69 |  | 67% | 13.69 | 9.61 |
| 17% | 6.73 | 14.65 |  | 68% | 13.77 | 9.45 |
| 18% | 6.93 | 14.61 |  | 69% | 13.85 | 9.29 |
| 19% | 7.13 | 14.57 |  | 70% | 13.92 | 9.12 |
| 20% | 7.32 | 14.52 |  | 71% | 13.99 | 8.95 |
| 21% | 7.51 | 14.47 |  | 72% | 14.06 | 8.78 |
| 22% | 7.70 | 14.42 |  | 73% | 14.13 | 8.61 |
| 23% | 7.89 | 14.37 |  | 74% | 14.19 | 8.43 |
| 24% | 8.07 | 14.31 |  | 75% | 14.25 | 8.25 |
| 25% | 8.25 | 14.25 |  | 76% | 14.31 | 8.07 |
| 26% | 8.43 | 14.19 |  | 77% | 14.37 | 7.89 |
| 27% | 8.61 | 14.13 |  | 78% | 14.42 | 7.70 |
| 28% | 8.78 | 14.06 |  | 79% | 14.47 | 7.51 |
| 29% | 8.95 | 13.99 |  | 80% | 14.52 | 7.32 |
| 30% | 9.12 | 13.92 |  | 81% | 14.57 | 7.13 |
| 31% | 9.29 | 13.85 |  | 82% | 14.61 | 6.93 |
| 32% | 9.45 | 13.77 |  | 83% | 14.65 | 6.73 |
| 33% | 9.61 | 13.69 |  | 84% | 14.69 | 6.53 |
| 34% | 9.77 | 13.61 |  | 85% | 14.73 | 6.33 |
| 35% | 9.93 | 13.53 |  | 86% | 14.76 | 6.12 |
| 36% | 10.08 | 13.44 |  | 87% | 14.80 | 5.92 |
| 37% | 10.24 | 13.36 |  | 88% | 14.83 | 5.71 |
| 38% | 10.39 | 13.27 |  | 89% | 14.85 | 5.49 |
| 39% | 10.53 | 13.17 |  | 90% | 14.88 | 5.28 |
| 40% | 10.68 | 13.08 |  | 91% | 14.90 | 5.06 |
| 41% | 10.82 | 12.98 |  | 92% | 14.92 | 4.84 |
| 42% | 10.96 | 12.88 |  | 93% | 14.94 | 4.62 |
| 43% | 11.10 | 12.78 |  | 94% | 14.96 | 4.40 |
| 44% | 11.24 | 12.68 |  | 95% | 14.97 | 4.17 |
| 45% | 11.37 | 12.57 |  | 96% | 14.98 | 3.94 |
| 46% | 11.50 | 12.46 |  | 97% | 14.99 | 3.71 |
| 47% | 11.63 | 12.35 |  | 98% | 15.00 | 3.48 |
| 48% | 11.76 | 12.24 |  | 99% | 15.00 | 3.24 |
| 49% | 11.88 | 12.12 |  | 100% | 15.00 | 3.00 |
| 50% | 12.00 | 12.00 |  |  |  |  |

**Option 2 (L=1.5)**

|  | **Payoff (in euros)**  **if number is** | |  |  | **Payoff (in euros)**  **if number** | |
| --- | --- | --- | --- | --- | --- | --- |
| **Probability judgment** | **In range** | **Outside of range** |  | **Probability judgment** | **In range** | **Outside of range** |
| 0% | 6.00 | 15.00 |  | 51% | 12.12 | 11.92 |
| 1% | 6.16 | 15.00 |  | 52% | 12.24 | 11.84 |
| 2% | 6.32 | 15.00 |  | 53% | 12.35 | 11.75 |
| 3% | 6.47 | 14.99 |  | 54% | 12.46 | 11.67 |
| 4% | 6.63 | 14.98 |  | 55% | 12.57 | 11.58 |
| 5% | 6.78 | 14.97 |  | 56% | 12.68 | 11.49 |
| 6% | 6.93 | 14.96 |  | 57% | 12.78 | 11.40 |
| 7% | 7.08 | 14.94 |  | 58% | 12.88 | 11.31 |
| 8% | 7.23 | 14.92 |  | 59% | 12.98 | 11.22 |
| 9% | 7.38 | 14.90 |  | 60% | 13.08 | 11.12 |
| 10% | 7.52 | 14.88 |  | 61% | 13.17 | 11.02 |
| 11% | 7.66 | 14.85 |  | 62% | 13.27 | 10.92 |
| 12% | 7.80 | 14.83 |  | 63% | 13.36 | 10.82 |
| 13% | 7.94 | 14.80 |  | 64% | 13.44 | 10.72 |
| 14% | 8.08 | 14.76 |  | 65% | 13.53 | 10.62 |
| 15% | 8.22 | 14.73 |  | 66% | 13.61 | 10.52 |
| 16% | 8.36 | 14.69 |  | 67% | 13.69 | 10.41 |
| 17% | 8.49 | 14.65 |  | 68% | 13.77 | 10.30 |
| 18% | 8.62 | 14.61 |  | 69% | 13.85 | 10.19 |
| 19% | 8.75 | 14.57 |  | 70% | 13.92 | 10.08 |
| 20% | 8.88 | 14.52 |  | 71% | 13.99 | 9.97 |
| 21% | 9.01 | 14.47 |  | 72% | 14.06 | 9.85 |
| 22% | 9.13 | 14.42 |  | 73% | 14.13 | 9.74 |
| 23% | 9.26 | 14.37 |  | 74% | 14.19 | 9.62 |
| 24% | 9.38 | 14.31 |  | 75% | 14.25 | 9.50 |
| 25% | 9.50 | 14.25 |  | 76% | 14.31 | 9.38 |
| 26% | 9.62 | 14.19 |  | 77% | 14.37 | 9.26 |
| 27% | 9.74 | 14.13 |  | 78% | 14.42 | 9.13 |
| 28% | 9.85 | 14.06 |  | 79% | 14.47 | 9.01 |
| 29% | 9.97 | 13.99 |  | 80% | 14.52 | 8.88 |
| 30% | 10.08 | 13.92 |  | 81% | 14.57 | 8.75 |
| 31% | 10.19 | 13.85 |  | 82% | 14.61 | 8.62 |
| 32% | 10.30 | 13.77 |  | 83% | 14.65 | 8.49 |
| 33% | 10.41 | 13.69 |  | 84% | 14.69 | 8.36 |
| 34% | 10.52 | 13.61 |  | 85% | 14.73 | 8.22 |
| 35% | 10.62 | 13.53 |  | 86% | 14.76 | 8.08 |
| 36% | 10.72 | 13.44 |  | 87% | 14.80 | 7.94 |
| 37% | 10.82 | 13.36 |  | 88% | 14.83 | 7.80 |
| 38% | 10.92 | 13.27 |  | 89% | 14.85 | 7.66 |
| 39% | 11.02 | 13.17 |  | 90% | 14.88 | 7.52 |
| 40% | 11.12 | 13.08 |  | 91% | 14.90 | 7.38 |
| 41% | 11.22 | 12.98 |  | 92% | 14.92 | 7.23 |
| 42% | 11.31 | 12.88 |  | 93% | 14.94 | 7.08 |
| 43% | 11.40 | 12.78 |  | 94% | 14.96 | 6.93 |
| 44% | 11.49 | 12.68 |  | 95% | 14.97 | 6.78 |
| 45% | 11.58 | 12.57 |  | 96% | 14.98 | 6.63 |
| 46% | 11.67 | 12.46 |  | 97% | 14.99 | 6.47 |
| 47% | 11.75 | 12.35 |  | 98% | 15.00 | 6.32 |
| 48% | 11.84 | 12.24 |  | 99% | 15.00 | 6.16 |
| 49% | 11.92 | 12.12 |  | 100% | 15.00 | 6.00 |
| 50% | 12.00 | 12.00 |  |  |  |  |

**Option 3 (L=2)**

|  | **Payoff (in euros)**  **if number is** | |  |  | **Payoff (in euros)**  **if number** | |
| --- | --- | --- | --- | --- | --- | --- |
| **Probability judgment** | **In range** | **Outside of range** |  | **Probability judgment** | **In range** | **Outside of range** |
| 0% | 7.50 | 15.00 |  | 51% | 12.12 | 11.94 |
| 1% | 7.62 | 15.00 |  | 52% | 12.24 | 11.88 |
| 2% | 7.74 | 15.00 |  | 53% | 12.35 | 11.81 |
| 3% | 7.85 | 14.99 |  | 54% | 12.46 | 11.75 |
| 4% | 7.97 | 14.98 |  | 55% | 12.57 | 11.69 |
| 5% | 8.09 | 14.97 |  | 56% | 12.68 | 11.62 |
| 6% | 8.20 | 14.96 |  | 57% | 12.78 | 11.55 |
| 7% | 8.31 | 14.94 |  | 58% | 12.88 | 11.48 |
| 8% | 8.42 | 14.92 |  | 59% | 12.98 | 11.41 |
| 9% | 8.53 | 14.90 |  | 60% | 13.08 | 11.34 |
| 10% | 8.64 | 14.88 |  | 61% | 13.17 | 11.27 |
| 11% | 8.75 | 14.85 |  | 62% | 13.27 | 11.19 |
| 12% | 8.85 | 14.83 |  | 63% | 13.36 | 11.12 |
| 13% | 8.96 | 14.80 |  | 64% | 13.44 | 11.04 |
| 14% | 9.06 | 14.76 |  | 65% | 13.53 | 10.97 |
| 15% | 9.17 | 14.73 |  | 66% | 13.61 | 10.89 |
| 16% | 9.27 | 14.69 |  | 67% | 13.69 | 10.81 |
| 17% | 9.37 | 14.65 |  | 68% | 13.77 | 10.73 |
| 18% | 9.47 | 14.61 |  | 69% | 13.85 | 10.64 |
| 19% | 9.56 | 14.57 |  | 70% | 13.92 | 10.56 |
| 20% | 9.66 | 14.52 |  | 71% | 13.99 | 10.48 |
| 21% | 9.76 | 14.47 |  | 72% | 14.06 | 10.39 |
| 22% | 9.85 | 14.42 |  | 73% | 14.13 | 10.30 |
| 23% | 9.94 | 14.37 |  | 74% | 14.19 | 10.21 |
| 24% | 10.03 | 14.31 |  | 75% | 14.25 | 10.13 |
| 25% | 10.13 | 14.25 |  | 76% | 14.31 | 10.03 |
| 26% | 10.21 | 14.19 |  | 77% | 14.37 | 9.94 |
| 27% | 10.30 | 14.13 |  | 78% | 14.42 | 9.85 |
| 28% | 10.39 | 14.06 |  | 79% | 14.47 | 9.76 |
| 29% | 10.48 | 13.99 |  | 80% | 14.52 | 9.66 |
| 30% | 10.56 | 13.92 |  | 81% | 14.57 | 9.56 |
| 31% | 10.64 | 13.85 |  | 82% | 14.61 | 9.47 |
| 32% | 10.73 | 13.77 |  | 83% | 14.65 | 9.37 |
| 33% | 10.81 | 13.69 |  | 84% | 14.69 | 9.27 |
| 34% | 10.89 | 13.61 |  | 85% | 14.73 | 9.17 |
| 35% | 10.97 | 13.53 |  | 86% | 14.76 | 9.06 |
| 36% | 11.04 | 13.44 |  | 87% | 14.80 | 8.96 |
| 37% | 11.12 | 13.36 |  | 88% | 14.83 | 8.85 |
| 38% | 11.19 | 13.27 |  | 89% | 14.85 | 8.75 |
| 39% | 11.27 | 13.17 |  | 90% | 14.88 | 8.64 |
| 40% | 11.34 | 13.08 |  | 91% | 14.90 | 8.53 |
| 41% | 11.41 | 12.98 |  | 92% | 14.92 | 8.42 |
| 42% | 11.48 | 12.88 |  | 93% | 14.94 | 8.31 |
| 43% | 11.55 | 12.78 |  | 94% | 14.96 | 8.20 |
| 44% | 11.62 | 12.68 |  | 95% | 14.97 | 8.09 |
| 45% | 11.69 | 12.57 |  | 96% | 14.98 | 7.97 |
| 46% | 11.75 | 12.46 |  | 97% | 14.99 | 7.85 |
| 47% | 11.81 | 12.35 |  | 98% | 15.00 | 7.74 |
| 48% | 11.88 | 12.24 |  | 99% | 15.00 | 7.62 |
| 49% | 11.94 | 12.12 |  | 100% | 15.00 | 7.50 |
| 50% | 12.00 | 12.00 |  |  |  |  |

**Option 4 (L=2.5)**

|  | **Payoff (in euros)**  **if number is** | |  |  | **Payoff (in euros)**  **if number** | |
| --- | --- | --- | --- | --- | --- | --- |
| **Probability judgment** | **In range** | **Outside of range** |  | **Probability judgment** | **In range** | **Outside of range** |
| 0% | 8.40 | 15.00 |  | 51% | 12.12 | 11.95 |
| 1% | 8.50 | 15.00 |  | 52% | 12.24 | 11.90 |
| 2% | 8.59 | 15.00 |  | 53% | 12.35 | 11.85 |
| 3% | 8.68 | 14.99 |  | 54% | 12.46 | 11.80 |
| 4% | 8.78 | 14.98 |  | 55% | 12.57 | 11.75 |
| 5% | 8.87 | 14.97 |  | 56% | 12.68 | 11.69 |
| 6% | 8.96 | 14.96 |  | 57% | 12.78 | 11.64 |
| 7% | 9.05 | 14.94 |  | 58% | 12.88 | 11.59 |
| 8% | 9.14 | 14.92 |  | 59% | 12.98 | 11.53 |
| 9% | 9.23 | 14.90 |  | 60% | 13.08 | 11.47 |
| 10% | 9.31 | 14.88 |  | 61% | 13.17 | 11.41 |
| 11% | 9.40 | 14.85 |  | 62% | 13.27 | 11.35 |
| 12% | 9.48 | 14.83 |  | 63% | 13.36 | 11.29 |
| 13% | 9.57 | 14.80 |  | 64% | 13.44 | 11.23 |
| 14% | 9.65 | 14.76 |  | 65% | 13.53 | 11.17 |
| 15% | 9.73 | 14.73 |  | 66% | 13.61 | 11.11 |
| 16% | 9.81 | 14.69 |  | 67% | 13.69 | 11.05 |
| 17% | 9.89 | 14.65 |  | 68% | 13.77 | 10.98 |
| 18% | 9.97 | 14.61 |  | 69% | 13.85 | 10.91 |
| 19% | 10.05 | 14.57 |  | 70% | 13.92 | 10.85 |
| 20% | 10.13 | 14.52 |  | 71% | 13.99 | 10.78 |
| 21% | 10.20 | 14.47 |  | 72% | 14.06 | 10.71 |
| 22% | 10.28 | 14.42 |  | 73% | 14.13 | 10.64 |
| 23% | 10.35 | 14.37 |  | 74% | 14.19 | 10.57 |
| 24% | 10.43 | 14.31 |  | 75% | 14.25 | 10.50 |
| 25% | 10.50 | 14.25 |  | 76% | 14.31 | 10.43 |
| 26% | 10.57 | 14.19 |  | 77% | 14.37 | 10.35 |
| 27% | 10.64 | 14.13 |  | 78% | 14.42 | 10.28 |
| 28% | 10.71 | 14.06 |  | 79% | 14.47 | 10.20 |
| 29% | 10.78 | 13.99 |  | 80% | 14.52 | 10.13 |
| 30% | 10.85 | 13.92 |  | 81% | 14.57 | 10.05 |
| 31% | 10.91 | 13.85 |  | 82% | 14.61 | 9.97 |
| 32% | 10.98 | 13.77 |  | 83% | 14.65 | 9.89 |
| 33% | 11.05 | 13.69 |  | 84% | 14.69 | 9.81 |
| 34% | 11.11 | 13.61 |  | 85% | 14.73 | 9.73 |
| 35% | 11.17 | 13.53 |  | 86% | 14.76 | 9.65 |
| 36% | 11.23 | 13.44 |  | 87% | 14.80 | 9.57 |
| 37% | 11.29 | 13.36 |  | 88% | 14.83 | 9.48 |
| 38% | 11.35 | 13.27 |  | 89% | 14.85 | 9.40 |
| 39% | 11.41 | 13.17 |  | 90% | 14.88 | 9.31 |
| 40% | 11.47 | 13.08 |  | 91% | 14.90 | 9.23 |
| 41% | 11.53 | 12.98 |  | 92% | 14.92 | 9.14 |
| 42% | 11.59 | 12.88 |  | 93% | 14.94 | 9.05 |
| 43% | 11.64 | 12.78 |  | 94% | 14.96 | 8.96 |
| 44% | 11.69 | 12.68 |  | 95% | 14.97 | 8.87 |
| 45% | 11.75 | 12.57 |  | 96% | 14.98 | 8.78 |
| 46% | 11.80 | 12.46 |  | 97% | 14.99 | 8.68 |
| 47% | 11.85 | 12.35 |  | 98% | 15.00 | 8.59 |
| 48% | 11.90 | 12.24 |  | 99% | 15.00 | 8.50 |
| 49% | 11.95 | 12.12 |  | 100% | 15.00 | 8.40 |
| 50% | 12.00 | 12.00 |  |  |  |  |

**Option 5 (L=3)**

|  | **Payoff (in euros)**  **if number is** | |  |  | **Payoff (in euros)**  **if number** | |
| --- | --- | --- | --- | --- | --- | --- |
| **Probability judgment** | **In range** | **Outside of range** |  | **Probability judgment** | **In range** | **Outside of range** |
| 0% | 9.00 | 15.00 |  | 51% | 12.12 | 11.96 |
| 1% | 9.08 | 15.00 |  | 52% | 12.24 | 11.92 |
| 2% | 9.16 | 15.00 |  | 53% | 12.35 | 11.88 |
| 3% | 9.24 | 14.99 |  | 54% | 12.46 | 11.83 |
| 4% | 9.31 | 14.98 |  | 55% | 12.57 | 11.79 |
| 5% | 9.39 | 14.97 |  | 56% | 12.68 | 11.75 |
| 6% | 9.47 | 14.96 |  | 57% | 12.78 | 11.70 |
| 7% | 9.54 | 14.94 |  | 58% | 12.88 | 11.65 |
| 8% | 9.61 | 14.92 |  | 59% | 12.98 | 11.61 |
| 9% | 9.69 | 14.90 |  | 60% | 13.08 | 11.56 |
| 10% | 9.76 | 14.88 |  | 61% | 13.17 | 11.51 |
| 11% | 9.83 | 14.85 |  | 62% | 13.27 | 11.46 |
| 12% | 9.90 | 14.83 |  | 63% | 13.36 | 11.41 |
| 13% | 9.97 | 14.80 |  | 64% | 13.44 | 11.36 |
| 14% | 10.04 | 14.76 |  | 65% | 13.53 | 11.31 |
| 15% | 10.11 | 14.73 |  | 66% | 13.61 | 11.26 |
| 16% | 10.18 | 14.69 |  | 67% | 13.69 | 11.20 |
| 17% | 10.24 | 14.65 |  | 68% | 13.77 | 11.15 |
| 18% | 10.31 | 14.61 |  | 69% | 13.85 | 11.10 |
| 19% | 10.38 | 14.57 |  | 70% | 13.92 | 11.04 |
| 20% | 10.44 | 14.52 |  | 71% | 13.99 | 10.98 |
| 21% | 10.50 | 14.47 |  | 72% | 14.06 | 10.93 |
| 22% | 10.57 | 14.42 |  | 73% | 14.13 | 10.87 |
| 23% | 10.63 | 14.37 |  | 74% | 14.19 | 10.81 |
| 24% | 10.69 | 14.31 |  | 75% | 14.25 | 10.75 |
| 25% | 10.75 | 14.25 |  | 76% | 14.31 | 10.69 |
| 26% | 10.81 | 14.19 |  | 77% | 14.37 | 10.63 |
| 27% | 10.87 | 14.13 |  | 78% | 14.42 | 10.57 |
| 28% | 10.93 | 14.06 |  | 79% | 14.47 | 10.50 |
| 29% | 10.98 | 13.99 |  | 80% | 14.52 | 10.44 |
| 30% | 11.04 | 13.92 |  | 81% | 14.57 | 10.38 |
| 31% | 11.10 | 13.85 |  | 82% | 14.61 | 10.31 |
| 32% | 11.15 | 13.77 |  | 83% | 14.65 | 10.24 |
| 33% | 11.20 | 13.69 |  | 84% | 14.69 | 10.18 |
| 34% | 11.26 | 13.61 |  | 85% | 14.73 | 10.11 |
| 35% | 11.31 | 13.53 |  | 86% | 14.76 | 10.04 |
| 36% | 11.36 | 13.44 |  | 87% | 14.80 | 9.97 |
| 37% | 11.41 | 13.36 |  | 88% | 14.83 | 9.90 |
| 38% | 11.46 | 13.27 |  | 89% | 14.85 | 9.83 |
| 39% | 11.51 | 13.17 |  | 90% | 14.88 | 9.76 |
| 40% | 11.56 | 13.08 |  | 91% | 14.90 | 9.69 |
| 41% | 11.61 | 12.98 |  | 92% | 14.92 | 9.61 |
| 42% | 11.65 | 12.88 |  | 93% | 14.94 | 9.54 |
| 43% | 11.70 | 12.78 |  | 94% | 14.96 | 9.47 |
| 44% | 11.75 | 12.68 |  | 95% | 14.97 | 9.39 |
| 45% | 11.79 | 12.57 |  | 96% | 14.98 | 9.31 |
| 46% | 11.83 | 12.46 |  | 97% | 14.99 | 9.24 |
| 47% | 11.88 | 12.35 |  | 98% | 15.00 | 9.16 |
| 48% | 11.92 | 12.24 |  | 99% | 15.00 | 9.08 |
| 49% | 11.96 | 12.12 |  | 100% | 15.00 | 9.00 |
| 50% | 12.00 | 12.00 |  |  |  |  |

**Option 6 (L=3.5)**

|  | **Payoff (in euros)**  **if number is** | |  |  | **Payoff (in euros)**  **if number** | |
| --- | --- | --- | --- | --- | --- | --- |
| **Probability judgment** | **In range** | **Outside of range** |  | **Probability judgment** | **In range** | **Outside of range** |
| 0% | 9.43 | 15.00 |  | 51% | 12.12 | 11.97 |
| 1% | 9.50 | 15.00 |  | 52% | 12.24 | 11.93 |
| 2% | 9.56 | 15.00 |  | 53% | 12.35 | 11.89 |
| 3% | 9.63 | 14.99 |  | 54% | 12.46 | 11.86 |
| 4% | 9.70 | 14.98 |  | 55% | 12.57 | 11.82 |
| 5% | 9.76 | 14.97 |  | 56% | 12.68 | 11.78 |
| 6% | 9.83 | 14.96 |  | 57% | 12.78 | 11.74 |
| 7% | 9.89 | 14.94 |  | 58% | 12.88 | 11.70 |
| 8% | 9.96 | 14.92 |  | 59% | 12.98 | 11.66 |
| 9% | 10.02 | 14.90 |  | 60% | 13.08 | 11.62 |
| 10% | 10.08 | 14.88 |  | 61% | 13.17 | 11.58 |
| 11% | 10.14 | 14.85 |  | 62% | 13.27 | 11.54 |
| 12% | 10.20 | 14.83 |  | 63% | 13.36 | 11.50 |
| 13% | 10.26 | 14.80 |  | 64% | 13.44 | 11.45 |
| 14% | 10.32 | 14.76 |  | 65% | 13.53 | 11.41 |
| 15% | 10.38 | 14.73 |  | 66% | 13.61 | 11.36 |
| 16% | 10.44 | 14.69 |  | 67% | 13.69 | 11.32 |
| 17% | 10.50 | 14.65 |  | 68% | 13.77 | 11.27 |
| 18% | 10.55 | 14.61 |  | 69% | 13.85 | 11.22 |
| 19% | 10.61 | 14.57 |  | 70% | 13.92 | 11.18 |
| 20% | 10.66 | 14.52 |  | 71% | 13.99 | 11.13 |
| 21% | 10.72 | 14.47 |  | 72% | 14.06 | 11.08 |
| 22% | 10.77 | 14.42 |  | 73% | 14.13 | 11.03 |
| 23% | 10.82 | 14.37 |  | 74% | 14.19 | 10.98 |
| 24% | 10.88 | 14.31 |  | 75% | 14.25 | 10.93 |
| 25% | 10.93 | 14.25 |  | 76% | 14.31 | 10.88 |
| 26% | 10.98 | 14.19 |  | 77% | 14.37 | 10.82 |
| 27% | 11.03 | 14.13 |  | 78% | 14.42 | 10.77 |
| 28% | 11.08 | 14.06 |  | 79% | 14.47 | 10.72 |
| 29% | 11.13 | 13.99 |  | 80% | 14.52 | 10.66 |
| 30% | 11.18 | 13.92 |  | 81% | 14.57 | 10.61 |
| 31% | 11.22 | 13.85 |  | 82% | 14.61 | 10.55 |
| 32% | 11.27 | 13.77 |  | 83% | 14.65 | 10.50 |
| 33% | 11.32 | 13.69 |  | 84% | 14.69 | 10.44 |
| 34% | 11.36 | 13.61 |  | 85% | 14.73 | 10.38 |
| 35% | 11.41 | 13.53 |  | 86% | 14.76 | 10.32 |
| 36% | 11.45 | 13.44 |  | 87% | 14.80 | 10.26 |
| 37% | 11.50 | 13.36 |  | 88% | 14.83 | 10.20 |
| 38% | 11.54 | 13.27 |  | 89% | 14.85 | 10.14 |
| 39% | 11.58 | 13.17 |  | 90% | 14.88 | 10.08 |
| 40% | 11.62 | 13.08 |  | 91% | 14.90 | 10.02 |
| 41% | 11.66 | 12.98 |  | 92% | 14.92 | 9.96 |
| 42% | 11.70 | 12.88 |  | 93% | 14.94 | 9.89 |
| 43% | 11.74 | 12.78 |  | 94% | 14.96 | 9.83 |
| 44% | 11.78 | 12.68 |  | 95% | 14.97 | 9.76 |
| 45% | 11.82 | 12.57 |  | 96% | 14.98 | 9.70 |
| 46% | 11.86 | 12.46 |  | 97% | 14.99 | 9.63 |
| 47% | 11.89 | 12.35 |  | 98% | 15.00 | 9.56 |
| 48% | 11.93 | 12.24 |  | 99% | 15.00 | 9.50 |
| 49% | 11.97 | 12.12 |  | 100% | 15.00 | 9.43 |
| 50% | 12.00 | 12.00 |  |  |  |  |

**Option 7 (L=4)**

|  | **Payoff (in euros)**  **if number is** | |  |  | **Payoff (in euros)**  **if number** | |
| --- | --- | --- | --- | --- | --- | --- |
| **Probability judgment** | **In range** | **Outside of range** |  | **Probability judgment** | **In range** | **Outside of range** |
| 0% | 9.75 | 15.00 |  | 51% | 12.12 | 11.97 |
| 1% | 9.81 | 15.00 |  | 52% | 12.24 | 11.94 |
| 2% | 9.87 | 15.00 |  | 53% | 12.35 | 11.91 |
| 3% | 9.93 | 14.99 |  | 54% | 12.46 | 11.88 |
| 4% | 9.99 | 14.98 |  | 55% | 12.57 | 11.84 |
| 5% | 10.04 | 14.97 |  | 56% | 12.68 | 11.81 |
| 6% | 10.10 | 14.96 |  | 57% | 12.78 | 11.78 |
| 7% | 10.16 | 14.94 |  | 58% | 12.88 | 11.74 |
| 8% | 10.21 | 14.92 |  | 59% | 12.98 | 11.71 |
| 9% | 10.27 | 14.90 |  | 60% | 13.08 | 11.67 |
| 10% | 10.32 | 14.88 |  | 61% | 13.17 | 11.63 |
| 11% | 10.37 | 14.85 |  | 62% | 13.27 | 11.60 |
| 12% | 10.43 | 14.83 |  | 63% | 13.36 | 11.56 |
| 13% | 10.48 | 14.80 |  | 64% | 13.44 | 11.52 |
| 14% | 10.53 | 14.76 |  | 65% | 13.53 | 11.48 |
| 15% | 10.58 | 14.73 |  | 66% | 13.61 | 11.44 |
| 16% | 10.63 | 14.69 |  | 67% | 13.69 | 11.40 |
| 17% | 10.68 | 14.65 |  | 68% | 13.77 | 11.36 |
| 18% | 10.73 | 14.61 |  | 69% | 13.85 | 11.32 |
| 19% | 10.78 | 14.57 |  | 70% | 13.92 | 11.28 |
| 20% | 10.83 | 14.52 |  | 71% | 13.99 | 11.24 |
| 21% | 10.88 | 14.47 |  | 72% | 14.06 | 11.19 |
| 22% | 10.92 | 14.42 |  | 73% | 14.13 | 11.15 |
| 23% | 10.97 | 14.37 |  | 74% | 14.19 | 11.11 |
| 24% | 11.02 | 14.31 |  | 75% | 14.25 | 11.06 |
| 25% | 11.06 | 14.25 |  | 76% | 14.31 | 11.02 |
| 26% | 11.11 | 14.19 |  | 77% | 14.37 | 10.97 |
| 27% | 11.15 | 14.13 |  | 78% | 14.42 | 10.92 |
| 28% | 11.19 | 14.06 |  | 79% | 14.47 | 10.88 |
| 29% | 11.24 | 13.99 |  | 80% | 14.52 | 10.83 |
| 30% | 11.28 | 13.92 |  | 81% | 14.57 | 10.78 |
| 31% | 11.32 | 13.85 |  | 82% | 14.61 | 10.73 |
| 32% | 11.36 | 13.77 |  | 83% | 14.65 | 10.68 |
| 33% | 11.40 | 13.69 |  | 84% | 14.69 | 10.63 |
| 34% | 11.44 | 13.61 |  | 85% | 14.73 | 10.58 |
| 35% | 11.48 | 13.53 |  | 86% | 14.76 | 10.53 |
| 36% | 11.52 | 13.44 |  | 87% | 14.80 | 10.48 |
| 37% | 11.56 | 13.36 |  | 88% | 14.83 | 10.43 |
| 38% | 11.60 | 13.27 |  | 89% | 14.85 | 10.37 |
| 39% | 11.63 | 13.17 |  | 90% | 14.88 | 10.32 |
| 40% | 11.67 | 13.08 |  | 91% | 14.90 | 10.27 |
| 41% | 11.71 | 12.98 |  | 92% | 14.92 | 10.21 |
| 42% | 11.74 | 12.88 |  | 93% | 14.94 | 10.16 |
| 43% | 11.78 | 12.78 |  | 94% | 14.96 | 10.10 |
| 44% | 11.81 | 12.68 |  | 95% | 14.97 | 10.04 |
| 45% | 11.84 | 12.57 |  | 96% | 14.98 | 9.99 |
| 46% | 11.88 | 12.46 |  | 97% | 14.99 | 9.93 |
| 47% | 11.91 | 12.35 |  | 98% | 15.00 | 9.87 |
| 48% | 11.94 | 12.24 |  | 99% | 15.00 | 9.81 |
| 49% | 11.97 | 12.12 |  | 100% | 15.00 | 9.75 |
| 50% | 12.00 | 12.00 |  |  |  |  |

**Option 8 (L=4.5)**

|  | **Payoff (in euros)**  **if number is** | |  |  | **Payoff (in euros)**  **if number** | |
| --- | --- | --- | --- | --- | --- | --- |
| **Probability judgment** | **In range** | **Outside of range** |  | **Probability judgment** | **In range** | **Outside of range** |
| 0% | 10.00 | 15.00 |  | 51% | 12.12 | 11.97 |
| 1% | 10.05 | 15.00 |  | 52% | 12.24 | 11.95 |
| 2% | 10.11 | 15.00 |  | 53% | 12.35 | 11.92 |
| 3% | 10.16 | 14.99 |  | 54% | 12.46 | 11.89 |
| 4% | 10.21 | 14.98 |  | 55% | 12.57 | 11.86 |
| 5% | 10.26 | 14.97 |  | 56% | 12.68 | 11.83 |
| 6% | 10.31 | 14.96 |  | 57% | 12.78 | 11.80 |
| 7% | 10.36 | 14.94 |  | 58% | 12.88 | 11.77 |
| 8% | 10.41 | 14.92 |  | 59% | 12.98 | 11.74 |
| 9% | 10.46 | 14.90 |  | 60% | 13.08 | 11.71 |
| 10% | 10.51 | 14.88 |  | 61% | 13.17 | 11.67 |
| 11% | 10.55 | 14.85 |  | 62% | 13.27 | 11.64 |
| 12% | 10.60 | 14.83 |  | 63% | 13.36 | 11.61 |
| 13% | 10.65 | 14.80 |  | 64% | 13.44 | 11.57 |
| 14% | 10.69 | 14.76 |  | 65% | 13.53 | 11.54 |
| 15% | 10.74 | 14.73 |  | 66% | 13.61 | 11.51 |
| 16% | 10.79 | 14.69 |  | 67% | 13.69 | 11.47 |
| 17% | 10.83 | 14.65 |  | 68% | 13.77 | 11.43 |
| 18% | 10.87 | 14.61 |  | 69% | 13.85 | 11.40 |
| 19% | 10.92 | 14.57 |  | 70% | 13.92 | 11.36 |
| 20% | 10.96 | 14.52 |  | 71% | 13.99 | 11.32 |
| 21% | 11.00 | 14.47 |  | 72% | 14.06 | 11.28 |
| 22% | 11.04 | 14.42 |  | 73% | 14.13 | 11.25 |
| 23% | 11.09 | 14.37 |  | 74% | 14.19 | 11.21 |
| 24% | 11.13 | 14.31 |  | 75% | 14.25 | 11.17 |
| 25% | 11.17 | 14.25 |  | 76% | 14.31 | 11.13 |
| 26% | 11.21 | 14.19 |  | 77% | 14.37 | 11.09 |
| 27% | 11.25 | 14.13 |  | 78% | 14.42 | 11.04 |
| 28% | 11.28 | 14.06 |  | 79% | 14.47 | 11.00 |
| 29% | 11.32 | 13.99 |  | 80% | 14.52 | 10.96 |
| 30% | 11.36 | 13.92 |  | 81% | 14.57 | 10.92 |
| 31% | 11.40 | 13.85 |  | 82% | 14.61 | 10.87 |
| 32% | 11.43 | 13.77 |  | 83% | 14.65 | 10.83 |
| 33% | 11.47 | 13.69 |  | 84% | 14.69 | 10.79 |
| 34% | 11.51 | 13.61 |  | 85% | 14.73 | 10.74 |
| 35% | 11.54 | 13.53 |  | 86% | 14.76 | 10.69 |
| 36% | 11.57 | 13.44 |  | 87% | 14.80 | 10.65 |
| 37% | 11.61 | 13.36 |  | 88% | 14.83 | 10.60 |
| 38% | 11.64 | 13.27 |  | 89% | 14.85 | 10.55 |
| 39% | 11.67 | 13.17 |  | 90% | 14.88 | 10.51 |
| 40% | 11.71 | 13.08 |  | 91% | 14.90 | 10.46 |
| 41% | 11.74 | 12.98 |  | 92% | 14.92 | 10.41 |
| 42% | 11.77 | 12.88 |  | 93% | 14.94 | 10.36 |
| 43% | 11.80 | 12.78 |  | 94% | 14.96 | 10.31 |
| 44% | 11.83 | 12.68 |  | 95% | 14.97 | 10.26 |
| 45% | 11.86 | 12.57 |  | 96% | 14.98 | 10.21 |
| 46% | 11.89 | 12.46 |  | 97% | 14.99 | 10.16 |
| 47% | 11.92 | 12.35 |  | 98% | 15.00 | 10.11 |
| 48% | 11.95 | 12.24 |  | 99% | 15.00 | 10.05 |
| 49% | 11.97 | 12.12 |  | 100% | 15.00 | 10.00 |
| 50% | 12.00 | 12.00 |  |  |  |  |

**Option 9 (L=5)**

|  | **Payoff (in euros)**  **if number is** | |  |  | **Payoff (in euros)**  **if number** | |
| --- | --- | --- | --- | --- | --- | --- |
| **Probability judgment** | **In range** | **Outside of range** |  | **Probability judgment** | **In range** | **Outside of range** |
| 0% | 10.20 | 15.00 |  | 51% | 12.12 | 11.98 |
| 1% | 10.25 | 15.00 |  | 52% | 12.24 | 11.95 |
| 2% | 10.30 | 15.00 |  | 53% | 12.35 | 11.93 |
| 3% | 10.34 | 14.99 |  | 54% | 12.46 | 11.90 |
| 4% | 10.39 | 14.98 |  | 55% | 12.57 | 11.87 |
| 5% | 10.43 | 14.97 |  | 56% | 12.68 | 11.85 |
| 6% | 10.48 | 14.96 |  | 57% | 12.78 | 11.82 |
| 7% | 10.52 | 14.94 |  | 58% | 12.88 | 11.79 |
| 8% | 10.57 | 14.92 |  | 59% | 12.98 | 11.76 |
| 9% | 10.61 | 14.90 |  | 60% | 13.08 | 11.74 |
| 10% | 10.66 | 14.88 |  | 61% | 13.17 | 11.71 |
| 11% | 10.70 | 14.85 |  | 62% | 13.27 | 11.68 |
| 12% | 10.74 | 14.83 |  | 63% | 13.36 | 11.65 |
| 13% | 10.78 | 14.80 |  | 64% | 13.44 | 11.62 |
| 14% | 10.82 | 14.76 |  | 65% | 13.53 | 11.59 |
| 15% | 10.87 | 14.73 |  | 66% | 13.61 | 11.55 |
| 16% | 10.91 | 14.69 |  | 67% | 13.69 | 11.52 |
| 17% | 10.95 | 14.65 |  | 68% | 13.77 | 11.49 |
| 18% | 10.99 | 14.61 |  | 69% | 13.85 | 11.46 |
| 19% | 11.03 | 14.57 |  | 70% | 13.92 | 11.42 |
| 20% | 11.06 | 14.52 |  | 71% | 13.99 | 11.39 |
| 21% | 11.10 | 14.47 |  | 72% | 14.06 | 11.36 |
| 22% | 11.14 | 14.42 |  | 73% | 14.13 | 11.32 |
| 23% | 11.18 | 14.37 |  | 74% | 14.19 | 11.29 |
| 24% | 11.21 | 14.31 |  | 75% | 14.25 | 11.25 |
| 25% | 11.25 | 14.25 |  | 76% | 14.31 | 11.21 |
| 26% | 11.29 | 14.19 |  | 77% | 14.37 | 11.18 |
| 27% | 11.32 | 14.13 |  | 78% | 14.42 | 11.14 |
| 28% | 11.36 | 14.06 |  | 79% | 14.47 | 11.10 |
| 29% | 11.39 | 13.99 |  | 80% | 14.52 | 11.06 |
| 30% | 11.42 | 13.92 |  | 81% | 14.57 | 11.03 |
| 31% | 11.46 | 13.85 |  | 82% | 14.61 | 10.99 |
| 32% | 11.49 | 13.77 |  | 83% | 14.65 | 10.95 |
| 33% | 11.52 | 13.69 |  | 84% | 14.69 | 10.91 |
| 34% | 11.55 | 13.61 |  | 85% | 14.73 | 10.87 |
| 35% | 11.59 | 13.53 |  | 86% | 14.76 | 10.82 |
| 36% | 11.62 | 13.44 |  | 87% | 14.80 | 10.78 |
| 37% | 11.65 | 13.36 |  | 88% | 14.83 | 10.74 |
| 38% | 11.68 | 13.27 |  | 89% | 14.85 | 10.70 |
| 39% | 11.71 | 13.17 |  | 90% | 14.88 | 10.66 |
| 40% | 11.74 | 13.08 |  | 91% | 14.90 | 10.61 |
| 41% | 11.76 | 12.98 |  | 92% | 14.92 | 10.57 |
| 42% | 11.79 | 12.88 |  | 93% | 14.94 | 10.52 |
| 43% | 11.82 | 12.78 |  | 94% | 14.96 | 10.48 |
| 44% | 11.85 | 12.68 |  | 95% | 14.97 | 10.43 |
| 45% | 11.87 | 12.57 |  | 96% | 14.98 | 10.39 |
| 46% | 11.90 | 12.46 |  | 97% | 14.99 | 10.34 |
| 47% | 11.93 | 12.35 |  | 98% | 15.00 | 10.30 |
| 48% | 11.95 | 12.24 |  | 99% | 15.00 | 10.25 |
| 49% | 11.98 | 12.12 |  | 100% | 15.00 | 10.20 |
| 50% | 12.00 | 12.00 |  |  |  |  |

**Option 10 (L=5.5)**

|  | **Payoff (in euros)**  **if number is** | |  |  | **Payoff (in euros)**  **if number** | |
| --- | --- | --- | --- | --- | --- | --- |
| **Probability judgment** | **In range** | **Outside of range** |  | **Probability judgment** | **In range** | **Outside of range** |
| 0% | 10.36 | 15.00 |  | 51% | 12.12 | 11.98 |
| 1% | 10.41 | 15.00 |  | 52% | 12.24 | 11.96 |
| 2% | 10.45 | 15.00 |  | 53% | 12.35 | 11.93 |
| 3% | 10.49 | 14.99 |  | 54% | 12.46 | 11.91 |
| 4% | 10.53 | 14.98 |  | 55% | 12.57 | 11.89 |
| 5% | 10.58 | 14.97 |  | 56% | 12.68 | 11.86 |
| 6% | 10.62 | 14.96 |  | 57% | 12.78 | 11.84 |
| 7% | 10.66 | 14.94 |  | 58% | 12.88 | 11.81 |
| 8% | 10.70 | 14.92 |  | 59% | 12.98 | 11.79 |
| 9% | 10.74 | 14.90 |  | 60% | 13.08 | 11.76 |
| 10% | 10.78 | 14.88 |  | 61% | 13.17 | 11.73 |
| 11% | 10.82 | 14.85 |  | 62% | 13.27 | 11.71 |
| 12% | 10.86 | 14.83 |  | 63% | 13.36 | 11.68 |
| 13% | 10.89 | 14.80 |  | 64% | 13.44 | 11.65 |
| 14% | 10.93 | 14.76 |  | 65% | 13.53 | 11.62 |
| 15% | 10.97 | 14.73 |  | 66% | 13.61 | 11.60 |
| 16% | 11.01 | 14.69 |  | 67% | 13.69 | 11.57 |
| 17% | 11.04 | 14.65 |  | 68% | 13.77 | 11.54 |
| 18% | 11.08 | 14.61 |  | 69% | 13.85 | 11.51 |
| 19% | 11.11 | 14.57 |  | 70% | 13.92 | 11.48 |
| 20% | 11.15 | 14.52 |  | 71% | 13.99 | 11.45 |
| 21% | 11.18 | 14.47 |  | 72% | 14.06 | 11.41 |
| 22% | 11.22 | 14.42 |  | 73% | 14.13 | 11.38 |
| 23% | 11.25 | 14.37 |  | 74% | 14.19 | 11.35 |
| 24% | 11.29 | 14.31 |  | 75% | 14.25 | 11.32 |
| 25% | 11.32 | 14.25 |  | 76% | 14.31 | 11.29 |
| 26% | 11.35 | 14.19 |  | 77% | 14.37 | 11.25 |
| 27% | 11.38 | 14.13 |  | 78% | 14.42 | 11.22 |
| 28% | 11.41 | 14.06 |  | 79% | 14.47 | 11.18 |
| 29% | 11.45 | 13.99 |  | 80% | 14.52 | 11.15 |
| 30% | 11.48 | 13.92 |  | 81% | 14.57 | 11.11 |
| 31% | 11.51 | 13.85 |  | 82% | 14.61 | 11.08 |
| 32% | 11.54 | 13.77 |  | 83% | 14.65 | 11.04 |
| 33% | 11.57 | 13.69 |  | 84% | 14.69 | 11.01 |
| 34% | 11.60 | 13.61 |  | 85% | 14.73 | 10.97 |
| 35% | 11.62 | 13.53 |  | 86% | 14.76 | 10.93 |
| 36% | 11.65 | 13.44 |  | 87% | 14.80 | 10.89 |
| 37% | 11.68 | 13.36 |  | 88% | 14.83 | 10.86 |
| 38% | 11.71 | 13.27 |  | 89% | 14.85 | 10.82 |
| 39% | 11.73 | 13.17 |  | 90% | 14.88 | 10.78 |
| 40% | 11.76 | 13.08 |  | 91% | 14.90 | 10.74 |
| 41% | 11.79 | 12.98 |  | 92% | 14.92 | 10.70 |
| 42% | 11.81 | 12.88 |  | 93% | 14.94 | 10.66 |
| 43% | 11.84 | 12.78 |  | 94% | 14.96 | 10.62 |
| 44% | 11.86 | 12.68 |  | 95% | 14.97 | 10.58 |
| 45% | 11.89 | 12.57 |  | 96% | 14.98 | 10.53 |
| 46% | 11.91 | 12.46 |  | 97% | 14.99 | 10.49 |
| 47% | 11.93 | 12.35 |  | 98% | 15.00 | 10.45 |
| 48% | 11.96 | 12.24 |  | 99% | 15.00 | 10.41 |
| 49% | 11.98 | 12.12 |  | 100% | 15.00 | 10.36 |
| 50% | 12.00 | 12.00 |  |  |  |  |

**Option 11 (L=6)**

|  | **Payoff (in euros)**  **if number is** | |  |  | **Payoff (in euros)**  **if number** | |
| --- | --- | --- | --- | --- | --- | --- |
| **Probability judgment** | **In range** | **Outside of range** |  | **Probability judgment** | **In range** | **Outside of range** |
| 0% | 10.50 | 15.00 |  | 51% | 12.12 | 11.98 |
| 1% | 10.54 | 15.00 |  | 52% | 12.24 | 11.96 |
| 2% | 10.58 | 15.00 |  | 53% | 12.35 | 11.94 |
| 3% | 10.62 | 14.99 |  | 54% | 12.46 | 11.92 |
| 4% | 10.66 | 14.98 |  | 55% | 12.57 | 11.90 |
| 5% | 10.70 | 14.97 |  | 56% | 12.68 | 11.87 |
| 6% | 10.73 | 14.96 |  | 57% | 12.78 | 11.85 |
| 7% | 10.77 | 14.94 |  | 58% | 12.88 | 11.83 |
| 8% | 10.81 | 14.92 |  | 59% | 12.98 | 11.80 |
| 9% | 10.84 | 14.90 |  | 60% | 13.08 | 11.78 |
| 10% | 10.88 | 14.88 |  | 61% | 13.17 | 11.76 |
| 11% | 10.92 | 14.85 |  | 62% | 13.27 | 11.73 |
| 12% | 10.95 | 14.83 |  | 63% | 13.36 | 11.71 |
| 13% | 10.99 | 14.80 |  | 64% | 13.44 | 11.68 |
| 14% | 11.02 | 14.76 |  | 65% | 13.53 | 11.66 |
| 15% | 11.06 | 14.73 |  | 66% | 13.61 | 11.63 |
| 16% | 11.09 | 14.69 |  | 67% | 13.69 | 11.60 |
| 17% | 11.12 | 14.65 |  | 68% | 13.77 | 11.58 |
| 18% | 11.16 | 14.61 |  | 69% | 13.85 | 11.55 |
| 19% | 11.19 | 14.57 |  | 70% | 13.92 | 11.52 |
| 20% | 11.22 | 14.52 |  | 71% | 13.99 | 11.49 |
| 21% | 11.25 | 14.47 |  | 72% | 14.06 | 11.46 |
| 22% | 11.28 | 14.42 |  | 73% | 14.13 | 11.43 |
| 23% | 11.31 | 14.37 |  | 74% | 14.19 | 11.40 |
| 24% | 11.34 | 14.31 |  | 75% | 14.25 | 11.38 |
| 25% | 11.38 | 14.25 |  | 76% | 14.31 | 11.34 |
| 26% | 11.40 | 14.19 |  | 77% | 14.37 | 11.31 |
| 27% | 11.43 | 14.13 |  | 78% | 14.42 | 11.28 |
| 28% | 11.46 | 14.06 |  | 79% | 14.47 | 11.25 |
| 29% | 11.49 | 13.99 |  | 80% | 14.52 | 11.22 |
| 30% | 11.52 | 13.92 |  | 81% | 14.57 | 11.19 |
| 31% | 11.55 | 13.85 |  | 82% | 14.61 | 11.16 |
| 32% | 11.58 | 13.77 |  | 83% | 14.65 | 11.12 |
| 33% | 11.60 | 13.69 |  | 84% | 14.69 | 11.09 |
| 34% | 11.63 | 13.61 |  | 85% | 14.73 | 11.06 |
| 35% | 11.66 | 13.53 |  | 86% | 14.76 | 11.02 |
| 36% | 11.68 | 13.44 |  | 87% | 14.80 | 10.99 |
| 37% | 11.71 | 13.36 |  | 88% | 14.83 | 10.95 |
| 38% | 11.73 | 13.27 |  | 89% | 14.85 | 10.92 |
| 39% | 11.76 | 13.17 |  | 90% | 14.88 | 10.88 |
| 40% | 11.78 | 13.08 |  | 91% | 14.90 | 10.84 |
| 41% | 11.80 | 12.98 |  | 92% | 14.92 | 10.81 |
| 42% | 11.83 | 12.88 |  | 93% | 14.94 | 10.77 |
| 43% | 11.85 | 12.78 |  | 94% | 14.96 | 10.73 |
| 44% | 11.87 | 12.68 |  | 95% | 14.97 | 10.70 |
| 45% | 11.90 | 12.57 |  | 96% | 14.98 | 10.66 |
| 46% | 11.92 | 12.46 |  | 97% | 14.99 | 10.62 |
| 47% | 11.94 | 12.35 |  | 98% | 15.00 | 10.58 |
| 48% | 11.96 | 12.24 |  | 99% | 15.00 | 10.54 |
| 49% | 11.98 | 12.12 |  | 100% | 15.00 | 10.50 |
| 50% | 12.00 | 12.00 |  |  |  |  |

**Option 12 (L=7)**

|  | **Payoff (in euros)**  **if number is** | |  |  | **Payoff (in euros)**  **if number** | |
| --- | --- | --- | --- | --- | --- | --- |
| **Probability judgment** | **In range** | **Outside of range** |  | **Probability judgment** | **In range** | **Outside of range** |
| 0% | 10.71 | 15.00 |  | 51% | 12.12 | 11.98 |
| 1% | 10.75 | 15.00 |  | 52% | 12.24 | 11.97 |
| 2% | 10.78 | 15.00 |  | 53% | 12.35 | 11.95 |
| 3% | 10.82 | 14.99 |  | 54% | 12.46 | 11.93 |
| 4% | 10.85 | 14.98 |  | 55% | 12.57 | 11.91 |
| 5% | 10.88 | 14.97 |  | 56% | 12.68 | 11.89 |
| 6% | 10.91 | 14.96 |  | 57% | 12.78 | 11.87 |
| 7% | 10.95 | 14.94 |  | 58% | 12.88 | 11.85 |
| 8% | 10.98 | 14.92 |  | 59% | 12.98 | 11.83 |
| 9% | 11.01 | 14.90 |  | 60% | 13.08 | 11.81 |
| 10% | 11.04 | 14.88 |  | 61% | 13.17 | 11.79 |
| 11% | 11.07 | 14.85 |  | 62% | 13.27 | 11.77 |
| 12% | 11.10 | 14.83 |  | 63% | 13.36 | 11.75 |
| 13% | 11.13 | 14.80 |  | 64% | 13.44 | 11.73 |
| 14% | 11.16 | 14.76 |  | 65% | 13.53 | 11.70 |
| 15% | 11.19 | 14.73 |  | 66% | 13.61 | 11.68 |
| 16% | 11.22 | 14.69 |  | 67% | 13.69 | 11.66 |
| 17% | 11.25 | 14.65 |  | 68% | 13.77 | 11.64 |
| 18% | 11.28 | 14.61 |  | 69% | 13.85 | 11.61 |
| 19% | 11.30 | 14.57 |  | 70% | 13.92 | 11.59 |
| 20% | 11.33 | 14.52 |  | 71% | 13.99 | 11.56 |
| 21% | 11.36 | 14.47 |  | 72% | 14.06 | 11.54 |
| 22% | 11.39 | 14.42 |  | 73% | 14.13 | 11.52 |
| 23% | 11.41 | 14.37 |  | 74% | 14.19 | 11.49 |
| 24% | 11.44 | 14.31 |  | 75% | 14.25 | 11.46 |
| 25% | 11.46 | 14.25 |  | 76% | 14.31 | 11.44 |
| 26% | 11.49 | 14.19 |  | 77% | 14.37 | 11.41 |
| 27% | 11.52 | 14.13 |  | 78% | 14.42 | 11.39 |
| 28% | 11.54 | 14.06 |  | 79% | 14.47 | 11.36 |
| 29% | 11.56 | 13.99 |  | 80% | 14.52 | 11.33 |
| 30% | 11.59 | 13.92 |  | 81% | 14.57 | 11.30 |
| 31% | 11.61 | 13.85 |  | 82% | 14.61 | 11.28 |
| 32% | 11.64 | 13.77 |  | 83% | 14.65 | 11.25 |
| 33% | 11.66 | 13.69 |  | 84% | 14.69 | 11.22 |
| 34% | 11.68 | 13.61 |  | 85% | 14.73 | 11.19 |
| 35% | 11.70 | 13.53 |  | 86% | 14.76 | 11.16 |
| 36% | 11.73 | 13.44 |  | 87% | 14.80 | 11.13 |
| 37% | 11.75 | 13.36 |  | 88% | 14.83 | 11.10 |
| 38% | 11.77 | 13.27 |  | 89% | 14.85 | 11.07 |
| 39% | 11.79 | 13.17 |  | 90% | 14.88 | 11.04 |
| 40% | 11.81 | 13.08 |  | 91% | 14.90 | 11.01 |
| 41% | 11.83 | 12.98 |  | 92% | 14.92 | 10.98 |
| 42% | 11.85 | 12.88 |  | 93% | 14.94 | 10.95 |
| 43% | 11.87 | 12.78 |  | 94% | 14.96 | 10.91 |
| 44% | 11.89 | 12.68 |  | 95% | 14.97 | 10.88 |
| 45% | 11.91 | 12.57 |  | 96% | 14.98 | 10.85 |
| 46% | 11.93 | 12.46 |  | 97% | 14.99 | 10.82 |
| 47% | 11.95 | 12.35 |  | 98% | 15.00 | 10.78 |
| 48% | 11.97 | 12.24 |  | 99% | 15.00 | 10.75 |
| 49% | 11.98 | 12.12 |  | 100% | 15.00 | 10.71 |
| 50% | 12.00 | 12.00 |  |  |  |  |
